# The Causal Effect of Supra‐ and Sub‐Gingival Instrumentation on the Oral Microbiome of Elderly Periodontitis Patients Treated in Nursing Homes: Secondary Analysis of a Randomised Controlled Trial

**DOI:** 10.1111/jcpe.70193

**Published:** 2026-08-19

**Authors:** Anna Greta Barbe, Daniel Hagenfeld, Carolin Walther, Dalia Beck, Fedja Farowski, Maria J. G. T. Vehreschild, Sonja H. M. Derman, Sven Kleine Bardenhorst, Dirk Bleiel

**Affiliations:** ^1^ Faculty of Medicine and University Hospital Cologne, Polyclinic for Operative Dentistry and Periodontology University of Cologne Cologne Germany; ^2^ Department of Periodontology and Operative Dentistry Münster University Hospital Münster Germany; ^3^ Department I of Internal Medicine University Hospital Cologne Cologne Germany; ^4^ Department II of Internal Medicine, Infectious Diseases Goethe University Frankfurt, University Hospital Frankfurt Frankfurt am Main Germany; ^5^ German Centre for Infection Research, Partner Site Bonn‐Cologne Cologne Germany; ^6^ Department of Clinical Epidemiology, Institute of Epidemiology and Social Medicine University of Münster Münster Germany; ^7^ Joint Dental Practice, Dres. Pia und Dirk Bleiel Rheinbreitbach Germany

**Keywords:** dental plaque, gingivitis, microbiome, nursing homes, periodontitis, supra‐ and sub‐gingival instrumentation

## Abstract

**Aims:**

Periodontitis among nursing home residents is increasing as more older adults retain their dentition. Evidence on the impact of periodontal therapy on their oral microbiota is limited. We aimed to assess the effect of outreach‐based supra‐ and sub‐gingival instrumentation on the periodontal microbiome.

**Materials and Methods:**

In this secondary analysis of an RCT, 36 residents (mean age 85 ± 6 years) with periodontitis had been randomised to supra‐ and sub‐gingival instrumentation (mean duration 14.3 ± 5.2 min) or a 3‐month control. Supragingival and subgingival plaque samples were collected at baseline and 3 months. Bacterial 16S rRNA sequencing and mixed‐effects modelling were used to assess alpha/beta diversity, dysbiosis index and differential abundance between groups over time.

**Results:**

The intervention group showed clinical improvements, with bleeding on probing decreasing from 45% ± 20% to 35% ± 16% and probing pocket depth decreasing from 4.5 ± 0.5 to 4.0 ± 0.5 mm. Dysbiosis was reduced compared with controls (*β* = −1.97, *p* = 0.0008). Beta‐diversity confirmed microbial shifts (*R* ^2^ = 0.0075, *p* = 0.025), characterised by decreases in differential abundance of *Treponema* and *Fretibacterium* and increases in *Lactobacillus* and *Actinomyces*.

**Conclusions:**

A pragmatic outreach‐based supra‐ and sub‐gingival instrumentation reduced microbial dysbiosis and improved periodontal parameters in institutionalised older adults, supporting its effectiveness in nursing home care.

**Trial Registration:**

German Clinical Trials Register (DRKS) registration number: DRKS00029392

## 1 Introduction

In industrialised nations, the proportion of older adults retaining their natural dentition has risen markedly over recent decades—a multifactorial testament to the success of preventive oral health programmes and advances in restorative and preventive dental care including fluoride exposure (Müller et al. 2017). As individuals live longer with more teeth, the burden of periodontitis among institutionalised older adults is increasing (Curtis et al. 2021). In nursing home populations, multimorbidity, polypharmacy, cognitive decline and restricted mobility contribute to a severely limited ability to perform oral hygiene independently or receive adequate assistance from nursing staff. This creates conditions conducive to oral dysbiosis and chronic inflammation (high levels of plaque, gingival bleeding, untreated periodontitis)—often resulting in progressive attachment loss, diminished quality of life (Csikar et al. 2025) and adverse outcomes such as pneumonia and frailty (Huang et al. 2025). The periodontal microbiome plays a pivotal role in the initiation and progression of periodontitis. These dysbiotic shifts are further amplified by age‐associated immune senescence and systemic diseases (Sarafidou et al. 2024); yet data on institutionalised populations remain scarce. Supra‐ and sub‐gingival instrumentation reduces the relative abundance of classical periodontitis‐associated taxa and partially re‐establishes the health‐associated microbiome (Baima et al. 2024; Krajewski et al. 2025). However, most trials involve middle‐aged patients and focus on pre–post changes in treated groups or on comparisons between different active treatment modalities, as in the case of systemic antibiotics (Teughels et al. 2020). Randomised comparisons against no periodontal treatment are rare—and virtually absent in institutionalised elderly populations.

Understanding microbial correlates of clinical improvement in nursing home residents may clarify the biological effectiveness of outreach periodontal care and ecological restoration of oral microbial communities. Accordingly, we performed a secondary microbiome analysis within a randomised controlled trial (RCT) of nursing home residents with periodontitis to evaluate the causal effect of short, single‐session, pragmatic supra‐ and sub‐gingival instrumentation delivered directly in the nursing home (Barbe et al. 2025) on the subgingival and supragingival microbiome. Residents were randomised either to supra‐ and sub‐gingival instrumentation (in accordance with the statutory health insurance guidelines in Germany (§22a SGB): combined supra‐ and sub‐gingival debridement) or to a waiting‐list control with no therapy for 3 months. This design mirrors real‐world delays in domiciliary dentistry and allows estimation of the causal effects of supra‐ and sub‐gingival instrumentation on periodontal microbial outcomes by comparing microbiome changes between treated and untreated residents.

## 2 Materials and Methods

### 2.1 Study Design and Ethical Approval

This microbiome investigation represents a pre‐specified secondary analysis of a previously registered RCT evaluating the effectiveness of outreach‐based periodontal therapy in nursing home residents (Barbe et al. 2025) (Trial registration: DRKS00029392, URL: https://www.drks.de/search/de/trial/DRKS00029392/details; 11‐02‐2023). The parent trial included 36 participants and evaluated clinical periodontal outcomes over a 3‐month period. Bleeding on probing (BOP) was the primary clinical endpoint of the parent study and formed the basis of the original sample size calculation. The present secondary analysis used the same randomised study population and follow‐up schedule to investigate microbiome‐related outcomes. Ethical approval was obtained from the Ethics Committee of the University of Cologne (22‐1219). All participants provided written informed consent for supragingival and subgingival microbial sampling. Further trial procedures (randomisation/allocation) followed the parent trial protocol (Supplementary methods in Supporting Information).

### 2.2 Participants and Intervention

Residents aged ≥ 65 years with periodontitis (probing pocket depth (PPD) ≥ 4 mm) and no systemic antibiotic use within the preceding 6 months were eligible (*N* = 36; 18 in control and 18 in intervention group). Exclusion criteria included medical conditions precluding safe periodontal instrumentation, anticipated short survival and antibiotic intake within 3 months before or during the study period.

The intervention consisted of a single‐session outreach supra‐ and sub‐gingival instrumentation delivered on‐site in the nursing home using a portable dental unit. All accessible teeth received supra‐ and sub‐gingival instrumentation (hand curettes and ultrasonic scalers), followed by polishing and pocket irrigation with 0.2% chlorhexidine. The intervention group received supra‐ and sub‐gingival instrumentation at baseline. Controls underwent identical baseline and 3‐month assessments without active periodontal treatment during the observation period and were offered supra‐ and sub‐gingival instrumentation after follow‐up.

### 2.3 Clinical Examination

At baseline and 3 months, a calibrated examiner (DiBl) recorded bleeding on probing (BOP), plaque index (PI) and pocket probing depth (PPD) at two sites per tooth (mesial and distal surfaces), using a periodontal probe (PCP 15, Hu‐Friedy, Chicago, IL, USA). Clinical attachment loss and radiographic bone loss were not assessed because the periodontal examination followed the pragmatic outreach care framework under German statutory health insurance regulations (§22a SGB V); therefore, formal staging and grading according to the current periodontitis classification could not be performed.

### 2.4 Plaque Sampling

Sub‐gingival and supragingival plaque samples were collected at baseline and 3 months. Sampling sites were defined at baseline as the teeth exhibiting the deepest periodontal pockets in each quadrant and were kept constant for longitudinal sampling, irrespective of changes in PPD over time. Supragingival biofilm was collected with a sterile curette. Sub‐gingival plaque was obtained from the deepest pocket sites in each quadrant with sterile ISO 25 paper points (15 s), then pooled, placed in DNA/RNA Shield (Zymo Research, Irvine, CA, USA) and stored at −80°C until extraction.

### 2.5 DNA Extraction, Sequencing and Bioinformatics

DNA was extracted using the ZymoBIOMICS DNA Miniprep Kit (Zymo Research). The V3–V4 region of the bacterial 16S rRNA gene was sequenced (Illumina NovaSeq, 2 × 250 bp, primers: 338F/806R). Reads were processed in QIIME 2 using DADA2 denoising to generate amplicon sequence variants; taxonomy was assigned using a Naïve Bayes classifier trained on eHOMD (RefSeq v16.01). Raw reads are available from ENA (PRJEB105287).

### 2.6 Microbiome Indices

Alpha‐diversity was summarised using standard within‐sample diversity indices (Richness, Shannon Index, Simpson Index), and beta‐diversity was quantified using between‐sample Bray–Curtis dissimilarity matrices (with constrained ordination used for visualisation) (Whittaker 1972). In addition, dysbiosis was summarised using the subgingival microbial dysbiosis index (SMDI). This was calculated as the mean centred log‐ratio abundance of periodontitis‐associated taxa minus the mean centred log‐ratio abundance of health‐associated taxa, such that higher values indicate greater dysbiosis (Chen et al. 2022).

### 2.7 Statistical Analysis

Baseline comparability and clinical changes over time were evaluated using nonparametric tests as appropriate (details are in Supplementary methods of Supporting Information). Microbiome outcomes were analysed longitudinally using mixed‐effects models accounting for repeated measures within participants, sampling niche (supragingival/subgingival) and sequencing depth. The primary microbiome estimand was the group × timepoint interaction, that is, differential change from baseline to 3 months between intervention and control arms. Baseline measurements were incorporated as part of the repeated‐measures structure of the mixed‐effects models rather than being entered as a separate covariate. We assessed (i) dysbiosis and alph‐ diversity, (ii) beta‐diversity and (iii) differential abundance with false discovery rate (FDR) control. Full model specifications (including filtering rules, permutation strategy, zero‐inflated negative‐binomial modelling, sensitivity analyses and the bootstrap‐based power analysis) are provided in the Supplementary methods of Supporting Information. The primary microbiome outcome of the present secondary analysis was the group × timepoint interaction for SMDI, representing the differential change in dysbiosis between intervention and control groups over time. Missing microbiome data were handled at the sample level. Analyses included all samples passing the predefined sequencing‐depth threshold of minimum 1000 reads. Because subgingival and supragingival samples were analysed separately, participants could contribute data from one site even if the paired sample was missing or excluded. All 36 residents had evaluable baseline microbiome data. At 3 months, one waiting‐list participant had no evaluable follow‐up sample, and one intervention participant had a supragingival follow‐up sample below the sequencing depth threshold, while the corresponding subgingival sample remained evaluable.

## 3 Results

### 3.1 Summary of Clinical Characteristics and Periodontal Clinical Findings

Thirty‐six nursing home residents were enrolled in the parent randomised controlled trial (mean age 84.8 ± 6.2 years; 75.0% female). There were no significant differences in demographic or oral health characteristics between the intervention and control groups at baseline (Table 1). Participants represented a highly vulnerable population, with a mean care level of 3.5 ± 0.8, a mean medication burden of 11.1 ± 4.0 active pharmaceutical ingredients and a dementia diagnosis present in 58.3% of participants. Oral hygiene dependency was common: while 55.6% of participants were able to perform oral hygiene independently, 41.7% required partial assistance and 2.8% were completely dependent on nursing staff.

**TABLE 1:** Baseline demographic, care‐related, and periodontal characteristics of the study population and principal clinical outcomes of the parent randomised controlled trial.

Characteristics	All (*N* = 36)	Intervention (*n* = 18)	Control (*n* = 18)	*p* ^a^
	Mean ± standard deviation (min.–max.) or *n* (%)			
Age (years)	84.8 ± 6.2	85.2 ± 5.6 (73–93)	84.5 ± 6.9 (69–95)	0.719
Active pharmaceutical ingredients^b^	11.1 ± 4	11.4 ± 3.4 (5–19)	10.8 ± 4.6 (6–20)	0.628
Care level	3.5 ± 0.8	3.8 ± 0.8 (3–5)	3.2 ± 0.8 (2–5)	0.085
Number of teeth	17 ± 7.5	17.4 ± 7.3 (6–30)	16.6 ± 7.9 (6–28)	0.767
Number of teeth with periodontal treatment need	15.6 ± 7.0	15.6 ± 6.8 (6–27)	15.6 ± 7.4 (3–27)	1.000
Sex				
Female	27 (75)	13 (72.2)	14 (77.8)	0.700^c^
Male	9 (25)	5 (27.8)	4 (22.2)	
Dementia diagnosis				
Yes	21 (58.3)	11 (61.1)	10 (55.6)	0.735^c^
No	15 (41.7)	7 (38.9)	8 (44.4)	
Oral hygiene independent	20 (55.6)	11 (61.1)	9 (50.0)	
Oral hygiene partially assisted	15 (41.7)	7 (38.9)	8 (44.4)	0.531^c^
Oral hygiene fully dependent	1 (2.8)	0	1 (5.6)	
Principal clinical periodontal parameters of the parent randomised controlled trial				
Baseline BOP (%)		45 ± 20	33 ± 25	0.059
3‐month BOP (%)		35 ± 16	38 ± 25^d^	
ΔBOP (%)		−10 ± 26	+5 ± 16	0.041
Baseline PPD (mm)		4.5 ± 0.5	4.3 ± 0.5	0.106
3‐month PPD (mm)		4.0 ± 0.5	4.3 ± 0.4^d^	
ΔPPD (mm)		−0.5 ± 0.5	+0.1 ± 0.4	< 0.001
Baseline PI		1.0 ± 0.4	0.8 ± 0.4	0.096
3‐month PI		0.7 ± 0.4	0.7 ± 0.4^d^	
ΔPI		−0.3 ± 0.4	−0.1 ± 0.3	0.090

*Note:* Data are presented as mean ± standard deviation (range) unless otherwise indicated.

Abbreviations: BOP, bleeding on probing; PI, plaque index; PPD, probing pocket depth.

^a^ Baseline *p*‐values represent between‐group comparisons at baseline (Mann–Whitney *U* test). *p*‐values reported for Δ values represent between‐group comparisons of changes from baseline to 3 months.

^b^ Active pharmaceutical ingredients: active components in a drug that produce the intended therapeutic effect.

^c^ Pearson Chi‐square test.

^d^ *n* = 17 at 3‐month follow‐up.

Participants retained a mean of 17.0 ± 7.5 natural teeth, of which 15.6 ± 7.0 (94.7% ± 10.3%) required periodontal treatment according to the German statutory treatment criteria. At baseline, the intervention group exhibited a mean BOP score of 45% ± 20%, a mean PPD of 4.5 ± 0.5 mm and a PI of 1.0 ± 0.4. Corresponding values in the control group were 33% ± 25%, 4.3 ± 0.5 mm and 0.8 ± 0.4, respectively, with no statistically significant between‐group differences (all *p* > 0.05).

The mean duration of outreach‐based supra‐ and sub‐gingival instrumentation was 14.3 ± 5.2 min. As previously reported in the parent trial, clinical periodontal parameters improved after treatment. Three months after intervention, BOP decreased from 45% ± 20% to 35% ± 16% (*p* = 0.041), PPD decreased from 4.5 ± 0.5 mm to 4.0 ± 0.5 mm (*p* < 0.001) and PI decreased from 1.0 ± 0.4 to 0.7 ± 0.4 in the intervention group but did not differ significantly between groups (*p* = 0.090) (Table 1).

### 3.2 Subgingival Microbial Dysbiosis Index and Alpha Diversity

Subgingival SMDI values were consistently higher than supragingival values across groups and timepoints. Mean paired differences ranged from 2.79 to 3.25 SMDI units and were comparable between groups (Figure 1). The group × timepoint interaction, adjusted for sequencing depth and participant‐specific random intercepts, was significant (*β* = −1.97 [95% CI: –3.09 to −0.84], *p* = 0.0008), indicating that changes in SMDI differed between arms (Figure 1). In the intervention arm, SMDI decreased from baseline to follow‐up (Δ = −1.01 SMDI units [95% CI: −1.80 to −0.21], *p* = 0.013), consistent with reduced dysbiosis. In contrast, the control arm showed an increase over time (Δ = +0.96 SMDI units [95% CI: 0.16 to 1.76], *p* = 0.020), yielding ~2.0‐unit between‐arm difference in change over time. SMDI was significantly lower in supragingival than in subgingival samples (*β* = −3.25 [95% CI: −3.89 to −2.61], *p* < 0.001). The library‐size covariate was not associated with SMDI (*β*_log(reads) = −0.08 [95% CI: −1.49 to 1.33], *p* = 0.91). Standard α‐diversity metrics (Richness, Shannon Index and Simpson Index) did not detect an intervention effect (Figure S1).

**FIGURE 1:**
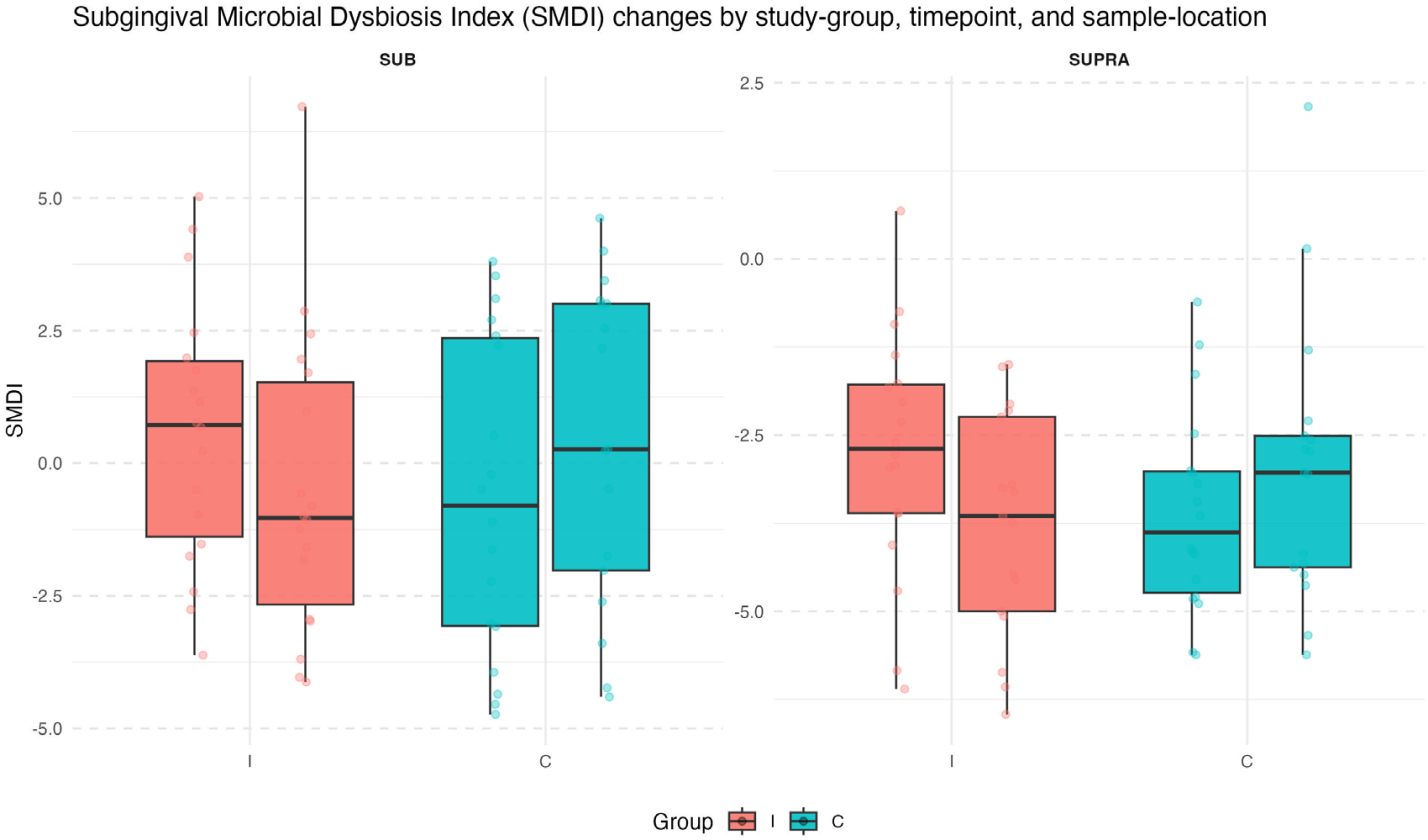
Subgingival microbial dysbiosis index (SMDI) stratified by plaque location, group (I = intervention, C = control) and timepoint (grouped bars). Boxes show medians and interquartile ranges; semi‐transparent points represent individual samples.

### 3.3 Beta Diversity

Using subject‐blocked restricted permutations, the group × timepoint interaction was significant (*R* ^2^ = 0.0075, *F* = 1.09, *p* = 0.025) after adjusting for plaque location and sequencing depth. This indicated a modest but detectable divergence in community trajectories between intervention and control over time. Plaque location was strongly associated with community composition (*R* ^2^ = 0.0453, *F* = 6.56, *p* = 0.005), and sequencing depth also showed a statistically noticeable association (*R* ^2^ = 0.0180, *F* = 2.61, *p* = 0.005). The PERMANOVA explained 9.0% of Bray–Curtis variation, with location accounting for 4.5%, sequencing depth for 1.8% and the group × timepoint interaction for 0.75%. The remaining 91.0% reflected inter‐individual heterogeneity and other factors not captured by the model. Constrained ordination supported the PERMANOVA results and illustrated the modest divergence in microbial trajectories between intervention and control groups over time (Figure 2).

**FIGURE 2:**
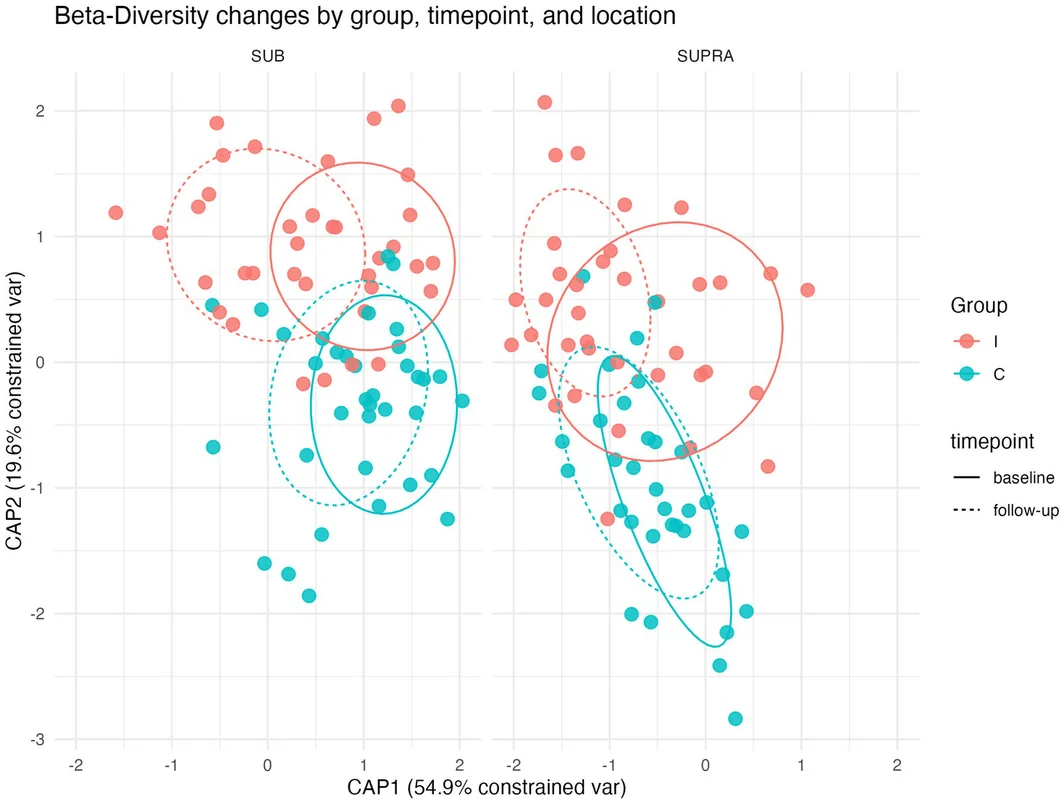
Constrained ordination of Bray–Curtis dissimilarities conditioned on location + timepoint × group (I = intervention, C = control) + log(read_depth). Points are labelled by group and timepoint; 95% ellipses highlight the modest directional shifts captured by the PERMANOVA interaction (partial *R* ^2^ ≈0.8%).

### 3.4 Differential Abundance

Differential abundance analysis showed consistent reductions in dysbiosis‐associated taxa, particularly *Treponema* and *Fretibacterium* lineages, together with increases in health‐associated taxa including *Lactobacillus*, *Neisseria*, *Granulicatella* and *Actinomyces* (see Table S1 and Figure 3). Species‐level analyses supported these findings, with significant decreases in *Treponema socranskii* and *Treponema vincentii* and increases in *Actinomyces naeslundii* , *Granulicatella adiacens* and *Abiotrophia defectiva* . These findings aligned with the SMDI analysis and indicated a shift away from anaerobic pathobiont‐rich profiles towards a more normobiotic community after intervention.

**FIGURE 3:**
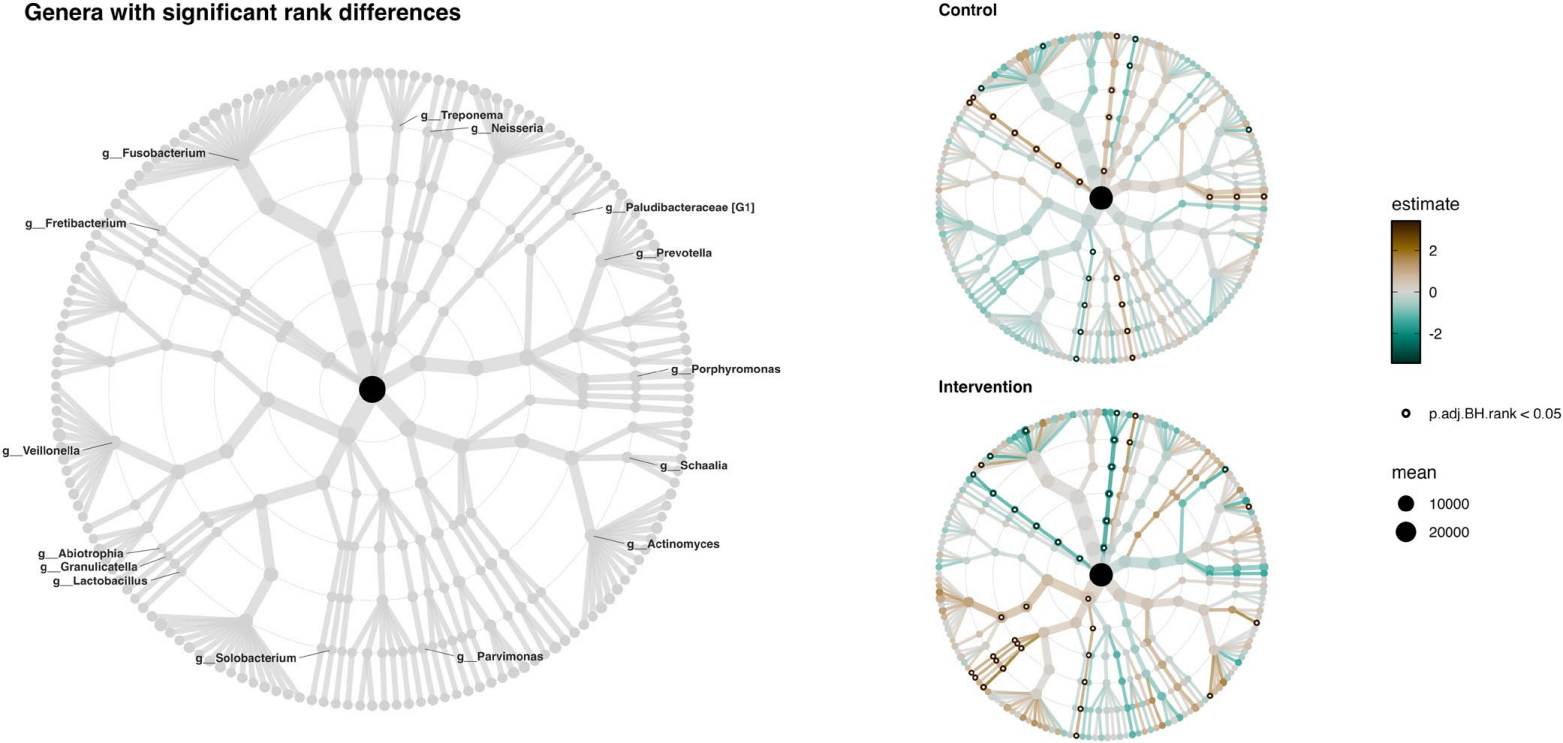
Taxonomic tree visualisation of differential rank shifts between study groups. The left panel highlights the genera annotated in the analysis and serves as a reference for taxon positions within the hierarchy. The right panels show effect estimates for Control (top) and Intervention (bottom) mapped onto the same taxonomic tree. Edge colour encodes the model estimate (legend: Brown colours indicate positive estimates and green colours indicate negative estimates) and node size is proportional to the mean abundance. Open circles indicate taxa meeting the multiple‐testing threshold (Benjamini–Hochberg‐adjusted rank‐based *p*‐value < 0.05).

### 3.5 Sensitivity Analysis

Comparing the estimates from stratified models for supragingival and subgingival sampling locations against the complete model showed that the SUPRA‐only model typically slightly shrank effects (median shift −0.28 log‐folds, interquartile range −0.57 to +0.08). In contrast, the SUB‐only model inflated them (median + 0.36, interquartile range +0.05 to +0.76). Significance patterns emphasised these magnitude differences: all 38 pooled‐model discoveries remained supported directionally in the stratified runs, but only four SUB terms (*Bacillota* and the *Lactobacillaceae* genus/family/species hierarchy) maintained *p*‐adj. < 0.05. No SUPRA‐only estimate crossed the FDR threshold, despite similar effect directions, likely reflecting smaller effect sizes and reduced statistical power after stratification (Table S1).

The baseline‐adjusted ANCOVA supported the primary longitudinal findings. In subgingival plaque, the intervention group had a lower 3‐month SMDI than the waiting‐list group after adjustment for baseline SMDI (adjusted difference −1.94, 95% CI: –3.41 to −0.48; *p* = 0.011) (Table S2).

## 4 Discussion

Our microbiome study in elderly nursing home residents provides the first randomised, controlled evidence that a single, short, pragmatic outreach‐based session of supra‐ and sub‐gingival instrumentation can reduce microbial dysbiosis and promote a shift towards a less dysbiotic microbial profile in older adults living in long‐term care. Despite advanced age, multimorbidity and compromised oral and systemic health, we observed a measurable ecological shift away from anaerobic proteolytic taxa associated with periodontal dysbiosis towards a less dysbiotic and more health‐associated microbial community. While SMDI values increased over time in untreated controls, the intervention group showed the opposite trajectory, resulting in a significant divergence between groups over the observation period. The observed divergence between groups was supported by the baseline‐adjusted sensitivity analysis, suggesting that the intervention contributed to the observed microbial changes.

### 4.1 Microbial Changes Following Simplified Periodontal Therapy

In contrast to the participants in the control arm, those receiving active therapy exhibited a pronounced reduction in the SMDI, consistent with a reduction in microbial dysbiosis. This bidirectional divergence underscores that in the absence of intervention, the control group exhibited a relative increase in dysbiosis over the observation period, although natural temporal variation cannot be excluded. The reduction of *Treponema* and *Fretibacterium* lineages, alongside the enrichment of normobiotic taxa such as *Lactobacillus* and *Actinomyces*, suggests that the ecological trajectory of periodontitis can be shifted towards a less dysbiotic state. These results align with RCTs investigating changes within the subgingival bacterial community before and after supra‐ and sub‐gingival instrumentation in much younger individuals (43.57 ± 6.63 years; Lu et al. 2022 and 34.95 ± 10.6 years; Nie et al. 2024). Among others, the relative abundance of *Treponema* and *Fretibacterium* decreased after supra‐ and sub‐gingival instrumentation, while the relative abundance of *Lactobacillus* and *Actinomyces* increased. Even though the magnitude of change was moderate compared with that in the younger cohorts, mechanical biofilm disruption can transiently reset microbial succession, independent of patients' age and therapy duration. Although subgingival sites exhibited substantially higher baseline dysbiosis, averaging effects across niches captured patient‐level ecological changes rather than site‐specific dynamics. In a stratified sensitivity analysis separately modelling supragingival and subgingival niches, the intervention × time interaction remained robust only in the subgingival compartment. In contrast, no supragingival taxon passed multiple‐testing correction when modelled alone—although effect directions were concordant with the combined analysis, suggesting limited statistical power rather than qualitative divergence.

Supragingival and subgingival plaque are anatomically adjacent and biologically connected, but they represent distinct ecological habitats (Welch Mark et al. 2020). Supragingival plaque is exposed to saliva, oxygen, dietary carbohydrates and daily oral hygiene, whereas subgingival plaque is influenced by the periodontal pocket environment, reduced oxygen tension, inflammatory exudate and host‐derived nutrients (Socransky and Haffajee 2002). In line with this, subgingival SMDI values were consistently higher than supragingival SMDI values, and location‐stratified taxon‐level analyses suggested stronger effect estimates in subgingival samples. Because SMDI was developed for subgingival plaque, we consider the subgingival SMDI findings the more biologically relevant dysbiosis endpoint, while supragingival analyses provide complementary information on broader plaque‐community changes.

### 4.2 Microbial–Clinical Concordance in a Frail Population

The observation of the previously reported clinical improvement (Barbe et al. 2025) and microbial reconfiguration supports the concept of ecological–clinical coupling between reductions in microbial dysbiosis and improvements in periodontal parameters. Reductions in PPD and BOP paralleled directional shifts towards a less dysbiotic community, indicating that compositional rebalancing may both reflect and reinforce decreased local inflammation. In frail and multimorbid elders—where immune senescence and systemic inflammation compromise repair (Sarafidou et al. 2024)—the oral microbiome retains its capacity for biological response, albeit in a diminished form. The absence of comparable improvements in controls (where no comparable improvements in SMDI or clinical indices were observed) strengthens the causal inference that the intervention, rather than stochastic fluctuation, drove the observed microbial and clinical improvements.

### 4.3 Constraints of Microbial Plasticity in Institutionalised Elders

While directionally favourable, the observed microbial and clinical changes remained moderate, reflecting the biological and environmental constraints of institutionalised ageing. Residents in long‐term care are chronically exposed to inflammatory stressors, salivary hypofunction, restricted diets and extensive polypharmacy—all of which may reduce microbial resilience and impede recolonisation by beneficial taxa (Deng et al. 2025). The participants were very old (mean age 85 years) and retained 17 natural teeth, reflecting current trends in tooth retention among older adults. Age‐related changes in oral ecology, tooth retention, immunosenescence and inflammaging may influence microbial community composition and partially explain why microbiological responses were less pronounced than those reported in younger populations.

Combined with limited oral hygiene capability and insufficient caregiver‐supported oral care (Asanza et al. 2025), these factors likely constrain the adaptive capacity of the oral microbiome and may explain why mechanical instrumentation alone resulted in only partial reductions in dysbiosis. This observation further highlights the potential importance of regular maintenance and supportive periodontal care in sustaining ecological and clinical improvements in frail older adults.

### 4.4 Intervention Feasibility and Real‐World Translation

The pragmatic design of this study reflects the realities of domiciliary dentistry, showing that measurable biological and clinical gains are achievable under the logistical and physical constraints of long‐term care. The single‐session outreach model—implemented with portable equipment and minimal pre‐treatment—proved feasible and safe, even for residents treated in wheelchairs or beds. Crucially, significant reductions in probing depth and dysbiosis were achieved without adjunctive antimicrobials or repeated maintenance visits. Beyond individual benefit, outreach periodontal programmes may contribute to improved access to oral health care among care‐dependent older adults. While the present study was not designed to assess systemic outcomes, evidence from other populations suggests that supra‐ and sub‐gingival instrumentation may reduce systemic inflammatory burden, as reflected by decreases in circulating inflammatory biomarkers following treatment, potentially lowering the risk of infection‐related systemic complications (Matsuda et al. 2024). However, whether comparable effects occur in very old and frail nursing home residents remains uncertain and warrants further investigation. Future outreach models may also benefit from teledentistry‐supported organisation, communication, education and monitoring strategies, which could facilitate access to oral health care and support preventive care in care‐dependent older adults.

### 4.5 Methodological Strengths and Limitations

The major strengths of this study include its randomised controlled design, real‐world implementation and dual‐niche microbiome assessment within a pre‐registered clinical trial framework. The use of mixed‐effects modelling with subject‐specific random intercepts, library‐size adjustments and FDR correction provided robust control for inter‐individual variation and sequencing heterogeneity. Integration of ecological metrics (SMDI, α‐ and *β*‐diversity) with taxon‐level differential abundance analysis allowed comprehensive characterisation of microbial change across scales. Nonetheless, certain limitations must be acknowledged. The modest sample size limited statistical power for subtle species‐level effects, and the 3‐month follow‐up captured only short‐term ecological perturbations. Because missingness was limited to one waiting‐list follow‐up participant and one low‐depth supragingival follow‐up sample, the impact on the mixed‐model analyses is expected to be small; nevertheless, we report the evaluable sample counts for transparency. Finally, as this trial was conducted within the German long‐term care system, generalisability beyond comparable long‐term care settings warrants cautious interpretation.

### 4.6 Future Directions and Clinical Implications

Future research should extend follow‐up to assess long‐term microbial stability and evaluate the effects of maintenance debridement, assisted hygiene and adjunctive therapeutics. Integrative approaches combining metagenomics, metatranscriptomics and host‐response profiling will be essential to uncover the molecular pathways linking microbial succession with inflammation resolution and tissue repair. The moderate magnitude of the observed microbiological and clinical improvements suggests that a single intervention is unlikely to provide long‐term ecological stability in this highly vulnerable population. Consequently, supportive periodontal therapy may be particularly important. In Germany, outreach maintenance care can be provided under §22a SGB V, which may facilitate preservation of the observed clinical and microbiological benefits. Clinically, the findings show that effective periodontal care does not require complex infrastructure: portable, outreach‐based treatment can deliver meaningful improvements even in frail elders, underscoring the ethical need for equitable, evidence‐based oral health care.

## 5 Conclusion

This RCT demonstrated that a single, pragmatic outreach‐based session of supra‐ and sub‐gingival instrumentation can elicit measurable clinical and microbial benefits even in frail institutionalised older adults. Importantly, supra‐ and sub‐gingival instrumentation promoted a shift towards a less dysbiotic microbial state and counteracted the progressive worsening of dysbiosis observed in untreated controls. Beyond clinical relevance, these results provide a translational framework for integrating domiciliary periodontal therapy into preventive geriatric care—bridging the gap between ecological understanding and equitable healthcare delivery for vulnerable ageing populations.

## Author Contributions

**Anna Greta Barbe:** conceptualisation, methodology, scientific supervision of data collection, data curation, formal analysis, statistical analysis, writing – original draft, writing – review and editing, data interpretation, supervision, validation of dental data and overall scientific quality assurance. **Daniel Hagenfeld:** microbial analysis, biomedical data analysis, figure development, writing – original draft, writing – review and editing. **Carolin Walther:** microbial analysis, biomedical data analysis, writing – review and editing. **Dalia Beck:** data collection, contribution to manuscript preparation. **Fedja Farowski:** sequencing and analysis of microbiological data, data evaluation, interpretation of results, critical review of the manuscript. **Maria J. G. T. Vehreschild:** sequencing and analysis of microbiological data, data evaluation, interpretation of results, critical review of the manuscript, quality assurance of microbiological analyses. **Sonja H. M. Derman:** investigation, project administration, quality control and implementation of study procedures, participant supervision during the study phase. **Sven Kleine Bardenhorst:** statistical and biomedical data analysis, interpretation of microbiological data, contribution to manuscript preparation. **Dirk Bleiel:** conceptualisation, methodology, data curation, writing – review and editing, data interpretation, supervision, validation of dental data and overall scientific quality assurance. All authors reviewed and approved the final version of the manuscript.

## Funding

The authors have nothing to report.

## Ethics Statement

The study received ethical approval from the local ethics review board of the University of Cologne, Cologne, Germany (22‐1219). All procedures complied with the ethical standards of the institutional research committee as well as the 1964 Helsinki Declaration and subsequent amendments or comparable ethical standards. Informed consent was obtained from all participants or their legal guardians.

## Conflicts of Interest

The authors declare no conflicts of interest.

## Supporting information


**Table S1:** Sensitivity analysis of effect estimates stratified by sampling location.

**Table S2:** Sensitivity analysis: Baseline‐adjusted ANCOVA model for follow‐up subgingival microbial dysbiosis index (SMDI).

**Data S1:** Supplementary methods.
**Figure S1:** Parametric bootstrap power curve for detecting the group × follow‐up interaction in the differential‐abundance pipeline. Power was estimated by simulating 150 datasets per candidate interaction effect (log *β*), using the fitted (zero‐inflated) negative‐binomial mixed model as the data‐generating process while preserving the original study design matrix, library‐size offsets, dispersion and subject‐level random intercepts. For each simulated dataset, the full workflow (taxon filtering, GLMM fitting and Benjamini–Hochberg correction within taxonomic rank) was rerun, and power was defined as the proportion of simulations in which the group × follow‐up term remained significant (BH *q* < 0.05).

## Data Availability

The data that support the findings of this study are openly available in The European Nucleotide Archive (ENA) at https://www.ebi.ac.uk/ena/, reference number PRJEB105287.
