# Supplementary material for: The Causal Effect of Supra‐ and Sub‐Gingival Instrumentation on the Oral Microbiome of Elderly Periodontitis Patients Treated in Nursing Homes: Secondary Analysis of a Randomised Controlled Trial
Source: J Clin Periodontol. 2026 Aug 19;53(12):1823–31. doi: 10.1111/jcpe.70193 (PMC13751316; doi:10.1111/jcpe.70193)
Supplement: Supplementary file 1 [file JCPE-53-1823-s001.docx]

**Supplementary Table 1: Sensitivity analysis of effect estimates stratified by sampling location**

| **rank** | **taxon** | **Estimate** **(Full)** | **FDR p-value** **(Full)** | **Estimate** **(SUPRA)** | **Estimate** **(SUB)** | **FDR p-value** **(SUPRA)** | **FDR p-value** **(SUB)** |
| --- | --- | --- | --- | --- | --- | --- | --- |
| Phylum | p__Actinomycetota | 0.26 | 0.64 | -0.07 | 0.64 | 0.9 | 0.48 |
| Phylum | p__Bacillota | 0.41 | **0.048** | 0.14 | 0.79 | 0.79 | **0.042** |
| Phylum | p__Bacteroidota | -0.32 | 0.42 | -0.12 | -0.43 | 0.88 |  |
| Phylum | p__Fusobacteriota | 0.04 | 0.94 | 0.22 | -0.02 | 0.86 | 0.96 |
| Phylum | p__Mycoplasmatota | -0.05 | 0.94 | -0.25 | 0.21 | 0.88 | 0.92 |
| Phylum | p__Patescibacteria | -0.14 | 0.92 | -2.09 | 1.41 | 0.091 | 0.19 |
| Phylum | p__Pseudomonadota | -0.24 | 0.58 | -0.24 | 0.19 | 0.86 | 0.83 |
| Phylum | p__Spirochaetota | -1.91 | **0.00053** | -0.75 | -1.07 | 0.79 | 0.3 |
| Phylum | p__Synergistota | -1.57 | **0.032** | -2.05 | -1.83 | 0.055 | 0.12 |
| Class | c__Actinomycetes | 0.45 | 0.41 | 0.24 | 0.68 | 0.74 | 0.43 |
| Class | c__Alphaproteobacteria | NA |  | NA | 1.38 |  | 0.31 |
| Class | c__Bacilli | 0.41 | 0.43 | 0.17 | 1.01 | 0.74 | 0.31 |
| Class | c__Bacteroidia | -0.42 | 0.41 | -0.26 | -0.49 | 0.74 | 0.55 |
| Class | c__Betaproteobacteria | 1.17 | 0.17 | 1.26 | 2.48 | 0.35 | 0.13 |
| Class | c__Clostridia | -0.33 | 0.48 | -0.68 | 0.33 | 0.34 | 0.56 |
| Class | c__Coriobacteriia | -0.54 | 0.56 | -2.05 | 0.31 | 0.1 | 0.88 |
| Class | c__Deltaproteobacteria | NA |  | NA | 0.44 |  | 0.74 |
| Class | c__Epsilonproteobacteria | -0.27 | 0.54 | -0.42 | -0.01 | 0.64 | 0.98 |
| Class | c__Erysipelotrichia | 1.38 | **0.032** | 0.94 | 1.74 | 0.53 | 0.11 |
| Class | c__Flavobacteriia | 1.34 | 0.1 | 1.13 | 1.00 | 0.47 | 0.55 |
| Class | c__Fusobacteriia | 0.04 | 0.92 | 0.22 | -0.02 | 0.74 | 0.98 |
| Class | c__Gammaproteobacteria | 0.15 | 0.9 | -0.95 | 0.43 | 0.52 | 0.8 |
| Class | c__Mollicutes | -0.05 | 0.95 | -0.25 | 0.21 | 0.86 | 0.89 |
| Class | c__Negativicutes | 0.68 | 0.052 | 0.37 | 0.84 | 0.54 | 0.33 |
| Class | c__Saccharimonadia | -0.14 | 0.9 | -2.09 | 1.41 | 0.097 | 0.29 |
| Class | c__Spirochaetia | -1.91 | **0.00071** | -0.75 | -1.07 | 0.64 | 0.33 |
| Class | c__Synergistia | -1.57 | **0.04** | -2.05 | -1.83 | 0.065 | 0.17 |
| Order | o__Actinomycetales | 0.29 | 0.66 | 0.05 | 0.76 | 0.95 | 0.44 |
| Order | o__Bacteroidales | -0.42 | 0.41 | -0.26 | -0.49 | 0.73 | 0.55 |
| Order | o__Bifidobacteriales | -0.08 | 0.94 | -0.55 | 0.76 | 0.73 | 0.62 |
| Order | o__Burkholderiales | NA |  | -0.30 | NA | 0.81 |  |
| Order | o__Campylobacterales | -0.27 | 0.61 | -0.42 | -0.01 | 0.64 | 0.98 |
| Order | o__Cardiobacteriales | NA |  | 1.07 | NA | 0.33 |  |
| Order | o__Caryophanales | -0.26 | 0.79 | -1.20 | 1.05 | 0.41 | 0.45 |
| Order | o__Coriobacteriales | -0.55 | 0.63 | -2.11 | 0.21 | 0.14 | 0.91 |
| Order | o__Desulfobacterales | NA |  | NA | 0.44 |  | 0.75 |
| Order | o__Eggerthellales | 0.17 | 0.87 | -0.90 | 1.59 | 0.52 | 0.16 |
| Order | o__Erysipelotrichales | 1.38 | **0.026** | 0.94 | 1.74 | 0.52 | 0.1 |
| Order | o__Eubacteriales | -0.76 | 0.26 | -1.56 | -0.75 | 0.096 | 0.55 |
| Order | o__Flavobacteriales | 1.34 | 0.09 | 1.13 | 1.00 | 0.41 | 0.55 |
| Order | o__Fusobacteriales | 0.04 | 0.93 | 0.22 | -0.02 | 0.78 | 0.97 |
| Order | o__Lachnospirales | -0.21 | 0.77 | -0.70 | 0.66 | 0.46 | 0.42 |
| Order | o__Lactobacillales | 0.44 | 0.41 | 0.20 | 1.01 | 0.73 | 0.34 |
| Order | o__Micrococcales | 1.02 | 0.24 | 0.88 | 1.76 | 0.46 | 0.25 |
| Order | o__Mycobacteriales | 0.32 | 0.73 | 0.47 | NA | 0.73 |  |
| Order | o__Mycoplasmoidales | -0.05 | 0.96 | -0.25 | 0.21 | 0.83 | 0.9 |
| Order | o__Neisseriales | 1.17 | 0.16 | 1.43 | 2.48 | 0.32 | 0.16 |
| Order | o__Pasteurellales | 0.15 | 0.91 | -1.14 | 0.43 | 0.41 | 0.82 |
| Order | o__Peptostreptococcales | -0.05 | 0.94 | 0.16 | -0.13 | 0.88 | 0.91 |
| Order | o__Rhodobacterales | NA |  | NA | 1.38 |  | 0.34 |
| Order | o__Saccharimonadales | -0.14 | 0.91 | -2.09 | 1.41 | 0.1 | 0.27 |
| Order | o__Selenomonadales | -0.21 | 0.77 | -0.48 | 0.20 | 0.67 | 0.87 |
| Order | o__Spirochaetales | -1.91 | **0.00059** | -0.75 | -1.07 | 0.66 | 0.4 |
| Order | o__Synergistales | -1.57 | **0.033** | -2.05 | -1.83 | 0.078 | 0.19 |
| Order | o__Tissierellales | -1.19 | 0.058 | -0.78 | -1.09 | 0.52 | 0.4 |
| Order | o__Veillonellales | 0.83 | **0.023** | 0.51 | 1.09 | 0.42 | 0.27 |
| Family | f__Actinomycetaceae | 0.29 | 0.69 | 0.05 | 0.76 | 0.98 | 0.45 |
| Family | f__Aerococcaceae | 1.36 | **0.023** | 0.88 | NA | 0.58 |  |
| Family | f__Anaerovoracaceae | -0.17 | 0.83 | -0.03 | -0.24 | 0.98 | 0.86 |
| Family | f__Atopobiaceae | -0.55 | 0.65 | -2.11 | 0.21 | 0.23 | 0.91 |
| Family | f__Bacillaceae | NA |  | NA | 0.95 |  | 0.57 |
| Family | f__Bacteroidaceae | -0.10 | 0.94 | -0.96 | -0.05 | 0.44 | 0.97 |
| Family | f__Bifidobacteriaceae | -0.08 | 0.95 | -0.55 | 0.76 | 0.77 | 0.66 |
| Family | f__Burkholderiaceae | NA |  | -0.30 | NA | 0.86 |  |
| Family | f__Campylobacteraceae | -0.27 | 0.62 | -0.42 | -0.01 | 0.69 | 0.98 |
| Family | f__Cardiobacteriaceae | NA |  | 1.07 | NA | 0.44 |  |
| Family | f__Carnobacteriaceae | 1.43 | **0.018** | 0.83 | 2.24 | 0.53 | 0.055 |
| Family | f__Corynebacteriaceae | 0.32 | 0.75 | 0.47 | NA | 0.77 |  |
| Family | f__Desulfobulbaceae | NA |  | NA | 0.44 |  | 0.8 |
| Family | f__Eggerthellaceae | 0.17 | 0.85 | -0.90 | 1.59 | 0.58 | 0.14 |
| Family | f__Enterococcaceae | NA |  | NA | 1.27 |  | 0.5 |
| Family | f__Erysipelotrichaceae | 1.38 | **0.026** | 0.94 | 1.74 | 0.58 | 0.056 |
| Family | f__Eubacteriaceae | -0.76 | 0.26 | -1.08 | -0.75 | 0.27 | 0.57 |
| Family | f__Flavobacteriaceae | 1.18 | 0.16 | 1.30 | 0.84 | 0.44 | 0.63 |
| Family | f__Fusobacteriaceae | 0.10 | 0.88 | 0.32 | -0.15 |  | 0.87 |
| Family | f__Gemellaceae | -0.26 | 0.79 | -1.20 | 0.64 | 0.47 | 0.63 |
| Family | f__Lachnospiraceae | -0.21 | 0.79 | -0.70 | 0.66 | 0.53 | 0.42 |
| Family | f__Lactobacillaceae | 2.03 | **0.0095** | 0.71 | 4.55 | 0.71 | **0.00017** |
| Family | f__Leptotrichiaceae | 0.21 | 0.83 | 0.09 | 0.27 | 0.98 | 0.87 |
| Family | f__Metamycoplasmataceae | -0.05 | 0.96 | -0.25 | 0.21 | 0.89 | 0.89 |
| Family | f__Micrococcaceae | 1.02 | 0.23 | 0.88 | 1.76 | 0.53 | 0.23 |
| Family | f__Nanoperiomorbaceae | -0.59 | 0.39 | -1.01 | 0.12 | 0.44 | 0.91 |
| Family | f__Neisseriaceae | 1.17 | 0.15 | 1.43 | 2.48 | 0.42 | 0.14 |
| Family | f__Paludibacteraceae | -1.03 | 0.33 | -0.16 | -2.06 | 0.98 | 0.2 |
| Family | f__Paracoccaceae | NA |  | NA | 1.38 |  | 0.32 |
| Family | f__Pasteurellaceae | 0.15 | 0.9 | -1.14 | 0.43 | 0.46 | 0.83 |
| Family | f__Peptoniphilaceae | -1.19 | 0.061 | -0.78 | -1.09 | 0.58 | 0.42 |
| Family | f__Peptostreptococcaceae | 0.64 | 0.37 | 0.42 | 1.16 | 0.77 | 0.32 |
| Family | f__Porphyromonadaceae | -1.03 | 0.13 | -1.35 | -0.74 | 0.27 | 0.63 |
| Family | f__Prevotellaceae | 0.18 | 0.79 | -0.19 | 0.57 | 0.86 | 0.57 |
| Family | f__Ruminococcaceae | NA |  | -0.72 | NA | 0.58 |  |
| Family | f__Saccharimonadaceae | 0.17 | 0.88 | -1.94 | 1.60 | 0.27 | 0.24 |
| Family | f__Selenomonadaceae | -0.21 | 0.79 | -0.48 | 0.20 | 0.71 | 0.86 |
| Family | f__Streptococcaceae | 0.16 | 0.79 | 0.16 | 0.32 | 0.86 | 0.82 |
| Family | f__Synergistaceae | -1.57 | **0.034** | -2.05 | -1.83 | 0.091 | 0.18 |
| Family | f__Tannerellaceae | -1.40 | 0.092 | -1.32 | -1.31 | 0.44 | 0.43 |
| Family | f__Treponemataceae | -1.91 | **0.00058** | -0.75 | -1.07 | 0.71 | 0.4 |
| Family | f__Veillonellaceae | 0.83 | **0.024** | 0.51 | 1.09 | 0.5 | 0.26 |
| Family | f__Weeksellaceae | 1.09 | 0.12 | 0.84 | 1.53 | 0.53 | 0.27 |
| Genus | g__Abiotrophia | 1.36 | **0.025** | 0.88 | NA | 0.6 |  |
| Genus | g__Actinomyces | 0.27 | 0.8 | 0.09 | 0.69 | 0.98 | 0.65 |
| Genus | g__Alloprevotella | 0.50 | 0.68 | 0.86 | 0.97 | 0.6 | 0.58 |
| Genus | g__Anaeroglobus | 0.45 | 0.65 | -0.38 | 0.79 | 0.79 | 0.46 |
| Genus | g__Anaerovoracaceae [G1] | 0.78 | 0.31 | 0.70 | 0.62 | 0.6 | 0.69 |
| Genus | g__Anaerovoracaceae [G4] | -0.80 | 0.15 | NA | -1.04 |  | 0.27 |
| Genus | g__Anoxybacillus | NA |  | NA | 0.95 |  | 0.62 |
| Genus | g__Bifidobacterium | -0.38 | 0.79 | -0.24 | 0.13 | 0.98 | 0.91 |
| Genus | g__Campylobacter | -0.27 | 0.68 | -0.42 | -0.01 | 0.72 | 0.98 |
| Genus | g__Capnocytophaga | 1.18 | 0.19 | 1.30 | 0.84 | 0.53 | 0.68 |
| Genus | g__Cardiobacterium | NA |  | 1.07 | NA | 0.53 |  |
| Genus | g__Catonella | -0.62 | 0.46 | -0.21 | 0.15 | 0.95 | 0.9 |
| Genus | g__Corynebacterium | 0.32 | 0.8 | 0.47 | NA | 0.8 |  |
| Genus | g__Cryptobacterium | 0.17 | 0.87 | -0.90 | 1.59 | 0.61 | 0.2 |
| Genus | g__Desulfobulbus | NA |  | NA | 0.44 |  | 0.83 |
| Genus | g__Dialister | 0.17 | 0.86 | 0.29 | 0.16 | 0.87 | 0.9 |
| Genus | g__Eikenella | NA |  | 1.43 | NA | 0.37 |  |
| Genus | g__Enterococcus | NA |  | NA | 1.27 |  | 0.54 |
| Genus | g__Fretibacterium | -1.57 | **0.04** | -2.05 | -1.83 | 0.17 | 0.25 |
| Genus | g__Fusobacterium | 0.10 | 0.9 | 0.32 | -0.15 |  | 0.9 |
| Genus | g__Gallibacter | -0.55 | 0.51 | -0.50 | -0.33 | 0.75 | 0.86 |
| Genus | g__Gemella | -0.26 | 0.84 | -1.20 | 0.64 | 0.58 | 0.68 |
| Genus | g__Granulicatella | 1.43 | **0.02** | 0.83 | 2.24 | 0.6 | 0.08 |
| Genus | g__Haematobacter | NA |  | NA | 1.38 |  | 0.35 |
| Genus | g__Haemophilus | 0.15 | 0.9 | -1.14 | 0.43 | 0.56 | 0.87 |
| Genus | g__Hallella | NA |  | NA | -1.36 |  | 0.25 |
| Genus | g__Hoylesella | -0.16 | 0.87 | -0.67 | 0.44 | 0.72 | 0.77 |
| Genus | g__Kingella | -0.82 | 0.34 | 0.39 | NA | 0.86 |  |
| Genus | g__Lachnoanaerobaculum | -0.51 | 0.66 | -0.46 | 1.20 | 0.72 | 0.43 |
| Genus | g__Lachnospiraceae [G7] | -0.10 | 0.9 | -0.64 | 0.06 | 0.67 | 0.94 |
| Genus | g__Lactobacillus | 2.28 | **0.0042** | 0.91 | 4.55 | 0.6 | **0.00024** |
| Genus | g__Lancefieldella | 1.47 | 0.077 | 0.19 | NA | 0.98 |  |
| Genus | g__Lautropia | NA |  | -0.30 | NA | 0.89 |  |
| Genus | g__Leptotrichia | 0.21 | 0.87 | 0.09 | 0.27 | 0.98 | 0.9 |
| Genus | g__Limosilactobacillus | 0.80 | 0.31 | 0.43 | NA | 0.8 |  |
| Genus | g__Megasphaera | NA |  | 0.44 | NA | 0.8 |  |
| Genus | g__Metamycoplasma | -0.05 | 0.96 | -0.25 | 0.21 | 0.93 | 0.9 |
| Genus | g__Nanoperiomorbus | -0.59 | 0.44 | -1.01 | 0.12 | 0.53 | 0.9 |
| Genus | g__Neisseria | 1.80 | **0.011** | 1.47 | 2.48 | 0.32 | 0.2 |
| Genus | g__Olsenella | -0.97 | 0.38 | -2.63 | 0.21 | 0.21 | 0.9 |
| Genus | g__Oribacterium | -0.15 | 0.87 | -0.29 | 0.38 | 0.89 | 0.82 |
| Genus | g__Paludibacteraceae [G1] | -1.03 | 0.37 | -0.16 | -2.06 | 0.98 | 0.26 |
| Genus | g__Parvimonas | -1.19 | 0.068 | -0.78 | -1.09 | 0.61 | 0.43 |
| Genus | g__Peptidiphaga | -0.12 | 0.92 | -0.89 | 0.70 | 0.6 | 0.8 |
| Genus | g__Peptostreptococcus | 0.64 | 0.41 | 0.42 | 1.16 | 0.8 | 0.35 |
| Genus | g__Phocaeicola | -0.10 | 0.94 | -0.96 | -0.05 | 0.53 | 0.97 |
| Genus | g__Porphyromonas | -1.03 | 0.15 | -1.35 | -0.74 | 0.32 | 0.68 |
| Genus | g__Prevotella | 0.02 | 0.97 | -0.28 | 0.45 | 0.87 | 0.82 |
| Genus | g__Pseudoramibacter | -0.76 | 0.31 | -1.08 | -0.75 | 0.32 | 0.62 |
| Genus | g__Riemerella | 1.09 | 0.14 | 0.84 | 1.53 | 0.6 | 0.31 |
| Genus | g__Rothia | 1.02 | 0.28 | 0.88 | 1.76 | 0.6 | 0.27 |
| Genus | g__Ruminococcaceae [G1] | NA |  | -1.19 | NA | 0.32 |  |
| Genus | g__Ruminococcaceae [G2] | NA |  | -0.11 | NA | 0.98 |  |
| Genus | g__Saccharimonas | 0.17 | 0.9 | -1.94 | 1.60 | 0.32 | 0.29 |
| Genus | g__Scardovia | 0.24 | 0.86 | -0.85 | 1.97 | 0.6 | 0.27 |
| Genus | g__Schaalia | 1.30 | 0.17 | 0.54 | 1.99 |  | 0.31 |
| Genus | g__Segatella | 0.58 | 0.55 | -0.20 | 1.51 | 0.97 | 0.33 |
| Genus | g__Selenomonadaceae [G1] | -0.14 | 0.9 | -0.90 | 0.21 |  | 0.9 |
| Genus | g__Selenomonas | -0.04 | 0.95 | -0.32 | 0.34 | 0.86 | 0.83 |
| Genus | g__Shuttleworthia | 0.05 | 0.95 | -0.74 | 0.98 | 0.72 | 0.46 |
| Genus | g__Solobacterium | 1.38 | **0.03** | 0.94 | 1.74 | 0.6 | 0.081 |
| Genus | g__Stomatobaculum | -0.30 | 0.84 | -1.08 | 1.36 | 0.6 | 0.46 |
| Genus | g__Streptococcus | 0.16 | 0.84 | 0.16 | 0.32 | 0.87 | 0.85 |
| Genus | g__Tannerella | -1.40 | 0.1 | -1.32 | -1.31 | 0.53 | 0.46 |
| Genus | g__Treponema | -1.91 | **0.00078** | -0.75 | -1.07 | 0.72 | 0.42 |
| Genus | g__Veillonella | 1.08 | 0.087 | 0.52 | 1.85 | 0.6 | 0.27 |
| Species | g__Fretibacterium Genus.70 | NA |  | NA | -1.12 |  | 0.33 |
| Species | g__Fretibacterium Genus.77 | -1.06 | 0.3 | -1.56 | -0.96 | 0.49 | 0.6 |
| Species | g__Fusobacterium Genus.386 | -0.31 | 0.82 | 0.15 | -1.15 | 0.98 | 0.52 |
| Species | g__Fusobacterium Genus.388 | NA |  | 0.86 | NA | 0.83 |  |
| Species | g__Fusobacterium Genus.391 | NA |  | 1.28 | NA | 0.58 |  |
| Species | g__Fusobacterium Genus.394 | -0.22 | 0.89 | -0.71 | 0.38 | 0.83 | 0.84 |
| Species | g__Fusobacterium Genus.404 | -0.97 | 0.4 | -0.88 | -0.62 | 0.79 | 0.77 |
| Species | g__Fusobacterium Genus.405 | -1.27 | 0.13 | -1.32 | -2.10 | 0.6 | 0.11 |
| Species | g__Fusobacterium Genus.407 | NA |  | NA | -1.68 |  | 0.45 |
| Species | g__Fusobacterium Genus.410 | 0.38 | 0.79 | -0.05 | 0.39 | 1 | 0.83 |
| Species | g__Fusobacterium Genus.411 | -0.46 | 0.66 | -1.03 | 0.44 | 0.58 | 0.83 |
| Species | g__Fusobacterium Genus.425 | NA |  | 1.52 | NA | 0.58 |  |
| Species | g__Fusobacterium Genus.432 | -0.48 | 0.76 | -1.07 | 0.04 | 0.74 | 0.98 |
| Species | g__Fusobacterium Genus.435 | -1.01 | 0.33 | -0.38 | -1.42 | 0.93 | 0.42 |
| Species | g__Haemophilus Genus.31 | NA |  | -1.39 | NA | 0.6 |  |
| Species | g__Lachnoanaerobaculum Genus.102 | -0.02 | 0.98 | -0.32 | NA | 0.93 |  |
| Species | g__Lachnoanaerobaculum Genus.76 | NA |  | -0.10 | NA | 0.98 |  |
| Species | g__Lactobacillus Genus | 2.28 | **0.0052** | 0.91 | 4.55 | 0.72 | **0.00063** |
| Species | g__Leptotrichia Genus.93 | NA |  | -0.19 | NA | 0.98 |  |
| Species | g__Neisseria Genus.71 | 1.80 | **0.013** | 1.44 | 2.48 | 0.55 | 0.3 |
| Species | g__Selenomonas Genus.203 | 0.08 | 0.93 | 0.26 | NA | 0.93 |  |
| Species | g__Streptococcus Genus.387 | 0.56 | 0.64 | 0.18 | NA | 0.98 |  |
| Species | g__Streptococcus Genus.388 | NA |  | 0.03 | NA | 1 |  |
| Species | g__Streptococcus Genus.389 | 0.11 | 0.94 | 0.01 | 0.80 | 1 | 0.72 |
| Species | g__Streptococcus Genus.393 | 0.48 | 0.65 | 0.22 | 1.08 | 0.94 | 0.52 |
| Species | g__Streptococcus Genus.564 | 0.15 | 0.91 | 0.35 | 0.06 | 0.92 | 0.98 |
| Species | g__Streptococcus Genus.566 | 0.01 | 0.99 | 0.19 | NA | 0.98 |  |
| Species | g__Streptococcus Genus.569 | -0.08 | 0.95 | -0.46 | 1.11 | 0.82 | 0.54 |
| Species | g__Streptococcus Genus.576 | NA |  | -0.12 | NA | 0.98 |  |
| Species | g__Veillonella Genus.254 | 0.77 | 0.37 | 0.12 | NA | 0.98 |  |
| Species | g__Veillonella Genus.259 | -0.07 | 0.96 | 0.20 | -0.32 | 0.98 | 0.84 |
| Species | g__Veillonella Genus.262 | 0.88 | 0.37 | 0.64 | NA | 0.83 |  |
| Species | g__Veillonella Genus.263 | 0.55 | 0.65 | 0.46 | 0.41 | 0.93 | 0.83 |
| Species | g__Veillonella Genus.265 | NA |  | 0.78 | NA | 0.78 |  |
| Species | g__Veillonella Genus.382 | -0.04 | 0.96 | -0.61 | 0.74 | 0.6 | 0.52 |
| Species | g__Veillonella Genus.386 | NA |  | -0.14 | NA | 0.98 |  |
| Species | s__abscessus.24 | -0.10 | 0.95 | -0.96 | -0.05 | 0.58 | 0.98 |
| Species | s__adiacens.33 | NA |  | NA | 0.51 |  | 0.71 |
| Species | s__adiacens.42 | 1.43 | **0.023** | 1.11 | 2.01 | 0.55 | 0.25 |
| Species | s__aeria.2 | NA |  | 0.18 | NA | 0.98 |  |
| Species | s__alactolyticus.25 | -0.76 | 0.33 | -1.08 | -0.75 | 0.49 | 0.67 |
| Species | s__anginosus.11 | 0.86 | 0.37 | 0.32 | NA | 0.93 |  |
| Species | s__anginosus.9 | -0.63 | 0.55 | 0.44 | -1.69 | 0.93 | 0.33 |
| Species | s__animalis | 0.20 | 0.89 | 0.61 | 0.15 | 0.84 | 0.92 |
| Species | s__animalis.1 | 1.62 | **0.034** | 1.56 | 0.67 | 0.58 | 0.72 |
| Species | s__animalis.22 | -0.71 | 0.57 | -0.43 | -0.74 | 0.93 | 0.72 |
| Species | s__animalis.23 | 0.79 | 0.44 | 0.72 | 0.24 | 0.85 | 0.86 |
| Species | s__animalis.6 | 1.53 | 0.1 | 1.36 | 1.68 | 0.58 | 0.39 |
| Species | s__animalis.7 | 0.76 | 0.42 | 0.67 | 1.12 | 0.83 | 0.42 |
| Species | s__atypica.30 | NA |  | -0.94 | NA | 0.63 |  |
| Species | s__atypica.41 | 0.39 | 0.68 | -0.40 | NA | 0.87 |  |
| Species | s__atypica.42 | NA |  | 0.63 | NA | 0.79 |  |
| Species | s__atypica.43 | 0.13 | 0.94 | -0.01 | NA | 1 |  |
| Species | s__bacterium HMT-075.11 | NA |  | -1.19 | NA | 0.49 |  |
| Species | s__bacterium HMT-085.23 | NA |  | -0.11 | NA | 0.98 |  |
| Species | s__bacterium HMT-086.4 | -0.10 | 0.92 | -0.64 | 0.06 | 0.78 | 0.97 |
| Species | s__bacterium HMT-103.7 | -0.80 | 0.17 | NA | -1.04 |  | 0.36 |
| Species | s__bacterium HMT-150.27 | 0.61 | 0.55 | -0.86 | 1.35 | 0.72 | 0.39 |
| Species | s__bacterium HMT-150.31 | -0.07 | 0.96 | -0.28 | 0.04 | 0.93 | 0.98 |
| Species | s__bacterium HMT-155.14 | -0.49 | 0.67 | -1.06 | -0.00 | 0.62 | 1 |
| Species | s__bacterium HMT-155.5 | -0.87 | 0.31 | -1.49 | -0.03 | 0.49 | 0.99 |
| Species | s__bacterium HMT-274.17 | 0.96 | 0.36 | -0.16 | NA | 0.98 |  |
| Species | s__bacterium HMT-274.21 | -1.72 | **0.046** | NA | -2.06 |  | 0.33 |
| Species | s__brachy.11 | -0.55 | 0.55 | -0.50 | -0.33 | 0.84 | 0.84 |
| Species | s__buccalis.9 | NA |  | -0.06 | NA | 0.99 |  |
| Species | s__cardiffensis.1 | NA |  | NA | 1.28 |  | 0.57 |
| Species | s__concisus.12 | -0.16 | 0.92 | -0.74 | NA | 0.83 |  |
| Species | s__concisus.2 | 0.76 | 0.35 | 0.12 | 0.93 | 0.98 | 0.57 |
| Species | s__concisus.3 | 0.64 | 0.35 | 0.87 | 0.53 | 0.46 | 0.73 |
| Species | s__constellatus.6 | -0.26 | 0.82 | 0.10 | 0.05 | 0.98 | 0.98 |
| Species | s__corrodens.2 | NA |  | 1.43 | NA | 0.51 |  |
| Species | s__curtum.4 | 0.17 | 0.89 | -0.90 | 1.59 | 0.74 | 0.3 |
| Species | s__defectiva.2 | 1.36 | **0.03** | 0.88 | NA | 0.72 |  |
| Species | s__dentalis.110 | NA |  | NA | -1.36 |  | 0.33 |
| Species | s__dentalis.68 | -0.24 | 0.89 | -0.48 | 0.29 | 0.93 | 0.89 |
| Species | s__dentalis.75 | -1.32 | 0.16 | -1.62 | -1.48 | 0.54 | 0.44 |
| Species | s__denticola.36 | -0.93 | 0.24 | -0.03 | -1.36 | 1 | 0.45 |
| Species | s__denticola.37 | -0.23 | 0.88 | -0.37 | -0.90 | 0.93 | 0.72 |
| Species | s__denticola.4 | -1.53 | 0.064 | NA | -1.48 |  | 0.4 |
| Species | s__dentium.25 | -0.38 | 0.77 | -0.24 | 0.13 | 0.98 | 0.93 |
| Species | s__dentocariosa.22 | 1.16 | 0.2 | 0.77 | 1.76 | 0.78 | 0.37 |
| Species | s__elegans.2 | NA |  | -1.05 | NA | 0.6 |  |
| Species | s__elongata.4 | NA |  | 1.07 | NA | 0.58 |  |
| Species | s__endodontalis.58 | NA |  | 0.09 | NA | 0.98 |  |
| Species | s__fastidiosum.11 | -1.77 | **0.0097** | -1.67 | -2.31 | 0.46 | 0.11 |
| Species | s__felix.50 | -0.02 | 0.98 | -0.37 | 0.67 | 0.93 | 0.74 |
| Species | s__fermentum.4 | 0.80 | 0.34 | 0.34 | NA | 0.9 |  |
| Species | s__flavithermus.4 | NA |  | NA | 0.95 |  | 0.67 |
| Species | s__flueggei.5 | NA |  | -0.44 | NA | 0.93 |  |
| Species | s__forsythia.26 | -1.40 | 0.12 | -2.26 | -1.31 | 0.12 | 0.52 |
| Species | s__geminatus.78 | 0.45 | 0.65 | -0.38 | 0.79 | 0.88 | 0.53 |
| Species | s__gerencseriae.10 | 0.29 | 0.73 | -0.20 | 1.06 | 0.96 | 0.37 |
| Species | s__gerencseriae.13 | 0.53 | 0.68 | 0.53 | 0.40 | 0.91 | 0.83 |
| Species | s__gerencseriae.5 | -0.05 | 0.97 | -0.91 | 1.31 | 0.72 | 0.38 |
| Species | s__gingivalis.43 | -1.03 | 0.17 | -1.35 | -0.74 | 0.49 | 0.72 |
| Species | s__gingivalis.92 | 0.72 | 0.49 | 0.58 | 0.84 | 0.79 | 0.72 |
| Species | s__gingivicola.7 | -0.06 | 0.96 | -0.43 | 0.62 | 0.9 | 0.72 |
| Species | s__gingivicola.8 | -0.13 | 0.89 | -0.22 | NA | 0.94 |  |
| Species | s__gordonii | 1.35 | 0.091 | 1.15 | 1.02 | 0.55 | 0.71 |
| Species | s__gracilis.144 | 0.29 | 0.85 | 0.31 | 0.48 | 0.95 | 0.83 |
| Species | s__gracilis.149 | -0.64 | 0.55 | -1.28 | -0.19 | 0.58 | 0.91 |
| Species | s__gracilis.155 | -1.10 | 0.27 | -0.49 | -1.61 | 0.92 | 0.38 |
| Species | s__gracilis.159 | 0.48 | 0.63 | 0.02 | NA | 1 |  |
| Species | s__gracilis.161 | 0.29 | 0.75 | 0.83 | NA | 0.66 |  |
| Species | s__granulosa.6 | NA |  | 0.73 | NA | 0.66 |  |
| Species | s__granulosa.7 | NA |  | 1.31 | NA | 0.58 |  |
| Species | s__haemolysans.3 | -0.26 | 0.84 | -1.20 | 0.64 | 0.62 | 0.72 |
| Species | s__hominis.14 | NA |  | 1.07 | NA | 0.58 |  |
| Species | s__hongkongensis.17 | 0.01 | 0.99 | -0.84 | NA | 0.79 |  |
| Species | s__hongkongensis.6 | 2.03 | **0.0024** | 2.09 | NA | 0.21 |  |
| Species | s__infirmum.9 | 0.78 | 0.34 | 0.70 | 0.62 | 0.72 | 0.73 |
| Species | s__intermedius.8 | NA |  | -0.20 | NA | 0.96 |  |
| Species | s__invisus.58 | 0.40 | 0.68 | 0.47 | 0.45 | 0.89 | 0.81 |
| Species | s__invisus.88 | -0.01 | 0.99 | 0.99 | -1.13 | 0.66 | 0.57 |
| Species | s__israelii.11 | 0.07 | 0.95 | NA | 0.16 |  | 0.91 |
| Species | s__israelii.37 | NA |  | -1.06 | NA | 0.58 |  |
| Species | s__italicus.17 | NA |  | NA | 1.27 |  | 0.57 |
| Species | s__leadbetteri.17 | NA |  | 0.49 | NA | 0.89 |  |
| Species | s__leadbetteri.23 | 0.53 | 0.66 | 0.82 | NA | 0.78 |  |
| Species | s__longum.11 | -0.30 | 0.83 | -1.08 | 1.36 | 0.66 | 0.52 |
| Species | s__maculosa.40 | 0.60 | 0.5 | 0.16 | 1.23 | 0.98 | 0.38 |
| Species | s__maltophilum.10 | -1.17 | 0.16 | NA | -1.48 |  | 0.33 |
| Species | s__maltophilum.12 | NA |  | NA | -1.06 |  | 0.45 |
| Species | s__massiliensis.6 | NA |  | 1.61 | NA | 0.53 |  |
| Species | s__matruchotii.33 | 0.32 | 0.79 | 0.47 | NA | 0.9 |  |
| Species | s__melaninogenica.30 | -0.08 | 0.95 | -0.84 | NA | 0.83 |  |
| Species | s__melaninogenica.41 | 1.43 | 0.084 | 1.56 | NA | 0.53 |  |
| Species | s__melaninogenica.46 | -0.27 | 0.84 | -0.31 | 0.64 | 0.94 | 0.78 |
| Species | s__melaninogenica.47 | -1.55 | 0.12 | -1.90 | -0.71 | 0.58 | 0.8 |
| Species | s__micra.9 | -1.19 | 0.081 | -0.78 | -1.09 | 0.77 | 0.5 |
| Species | s__micronuciformis.21 | NA |  | 0.44 | NA | 0.89 |  |
| Species | s__mirabilis.20 | NA |  | -0.30 | NA | 0.93 |  |
| Species | s__missouriensis.3 | NA |  | NA | 1.38 |  | 0.42 |
| Species | s__mitis.1 | 0.97 | 0.18 | 0.69 | 1.38 | 0.74 | 0.39 |
| Species | s__moorei.14 | 1.38 | **0.035** | 0.94 | 1.74 | 0.73 | 0.11 |
| Species | s__morbi.1 | -0.62 | 0.5 | -0.21 | -0.59 | 0.96 | 0.72 |
| Species | s__mucilaginosa.56 | 0.24 | 0.87 | -1.10 | NA | 0.73 |  |
| Species | s__mutans.11 | 0.99 | 0.37 | 1.32 | 1.03 | 0.58 | 0.62 |
| Species | s__naeslundii.3 | 0.70 | 0.55 | 0.79 | NA | 0.74 |  |
| Species | s__naeslundii.5 | 1.61 | **0.038** | 1.21 | NA | 0.58 |  |
| Species | s__nigrescens.15 | 0.32 | 0.82 | -0.40 | 0.47 | 0.93 | 0.78 |
| Species | s__nigrescens.16 | 0.07 | 0.95 | 0.29 | 0.38 | 0.93 | 0.83 |
| Species | s__orale.29 | NA |  | -1.15 | NA | 0.58 |  |
| Species | s__oralis.115 | -0.16 | 0.89 | -0.67 | 0.44 | 0.83 | 0.77 |
| Species | s__oralis.42 | -0.82 | 0.37 | 0.39 | NA | 0.92 |  |
| Species | s__oralis.6 | NA |  | 0.29 | NA | 0.93 |  |
| Species | s__oralis.95 | NA |  | NA | 0.44 |  | 0.83 |
| Species | s__oris.101 | 0.47 | 0.65 | 0.43 | 1.55 | 0.92 | 0.42 |
| Species | s__oris.113 | 0.97 | 0.23 | 0.51 | 1.66 | 0.9 | 0.33 |
| Species | s__oris.12 | 0.74 | 0.6 | -0.62 | 2.90 | 0.9 | 0.11 |
| Species | s__oris.13 | NA |  | 1.61 | NA | 0.58 |  |
| Species | s__oris.139 | NA |  | NA | -0.47 |  | 0.83 |
| Species | s__oris.15 | NA |  | 0.36 | NA | 0.92 |  |
| Species | s__oris.9 | 1.61 | 0.075 | 1.84 | NA | 0.58 |  |
| Species | s__parainfluenzae.31 | 0.15 | 0.93 | -0.42 | 0.43 | 0.92 | 0.84 |
| Species | s__parainfluenzae.37 | NA |  | 0.68 | NA | 0.79 |  |
| Species | s__parainfluenzae.46 | NA |  | -1.17 | NA | 0.51 |  |
| Species | s__parasanguinis | 1.05 | 0.12 | 0.49 | NA | 0.89 |  |
| Species | s__parasanguinis.3 | 1.41 | 0.19 | 0.58 | NA | 0.9 |  |
| Species | s__parvula.56 | 1.47 | 0.093 | 0.19 | NA | 0.98 |  |
| Species | s__parvula.79 | NA |  | -0.29 | NA | 0.93 |  |
| Species | s__parvula.81 | -0.06 | 0.96 | -0.22 | NA | 0.94 |  |
| Species | s__parvula.83 | -0.04 | 0.97 | -0.12 | NA | 0.98 |  |
| Species | s__parvula.86 | 1.09 | 0.15 | 0.72 | 1.63 | 0.59 | 0.38 |
| Species | s__parvula.96 | 0.92 | 0.26 | 0.43 | 1.87 | 0.84 | 0.37 |
| Species | s__parvula.97 | 1.54 | 0.064 | 1.64 | 2.49 | 0.55 | 0.33 |
| Species | s__periodonticus.12 | -0.59 | 0.47 | -1.01 | 0.12 | 0.59 | 0.92 |
| Species | s__pneumosintes.13 | -0.40 | 0.67 | -0.92 | 0.27 | 0.6 | 0.84 |
| Species | s__pneumosintes.7 | 0.61 | 0.35 | 1.20 | 0.38 | 0.49 | 0.74 |
| Species | s__polymorphum.10 | 0.55 | 0.7 | -0.12 | 0.76 | 0.98 | 0.78 |
| Species | s__polymorphum.8 | 0.67 | 0.66 | 0.76 | NA | 0.87 |  |
| Species | s__reuteri.8 | NA |  | 0.72 | NA | 0.77 |  |
| Species | s__saburreum.113 | -0.60 | 0.57 | -1.13 | 1.20 | 0.51 | 0.5 |
| Species | s__salivae.39 | NA |  | -1.11 | NA | 0.71 |  |
| Species | s__salivae.79 | 0.30 | 0.84 | -1.09 | 1.12 | 0.74 | 0.57 |
| Species | s__salivarium.8 | -0.05 | 0.97 | -0.25 | 0.21 | 0.95 | 0.9 |
| Species | s__salivarius.8 | 0.88 | 0.44 | 0.41 | 1.20 | 0.91 | 0.53 |
| Species | s__sanguinis.16 | NA |  | -0.17 | NA | 0.98 |  |
| Species | s__sanguinis.17 | NA |  | -0.46 | NA | 0.87 |  |
| Species | s__satelles.16 | 0.05 | 0.97 | -0.74 | 0.98 | 0.82 | 0.53 |
| Species | s__serpentiformis.7 | NA |  | -0.16 | NA | 0.98 |  |
| Species | s__showae.11 | -0.51 | 0.67 | -0.15 | -0.68 | 0.98 | 0.72 |
| Species | s__showae.2 | -0.91 | 0.55 | -1.03 | -1.08 | 0.81 | 0.69 |
| Species | s__socranskii.13 | NA |  | NA | 0.93 |  | 0.57 |
| Species | s__socranskii.21 | NA |  | NA | 0.87 |  | 0.61 |
| Species | s__socranskii.35 | -0.30 | 0.83 | NA | 0.05 |  | 0.98 |
| Species | s__socranskii.36 | NA |  | NA | -1.75 |  | 0.083 |
| Species | s__socranskii.6 | -1.25 | 0.059 | -0.73 | -1.10 | 0.83 | 0.38 |
| Species | s__socranskii.9 | -1.68 | **0.017** | -0.74 | -2.32 | 0.82 | 0.11 |
| Species | s__sp. HMT-036.5 | NA |  | -1.45 | NA | 0.46 |  |
| Species | s__sp. HMT-036.7 | NA |  | -2.58 | NA | 0.12 |  |
| Species | s__sp. HMT-078.65 | -0.15 | 0.89 | -0.29 | 0.38 | 0.93 | 0.82 |
| Species | s__sp. HMT-164.17 | NA |  | NA | 1.10 |  | 0.52 |
| Species | s__sp. HMT-180.10 | 0.18 | 0.92 | -0.37 | 0.65 | 0.97 | 0.75 |
| Species | s__sp. HMT-180.9 | 0.44 | 0.7 | 0.01 | 1.81 | 1 | 0.38 |
| Species | s__sp. HMT-183.11 | -0.21 | 0.9 | -0.97 | 0.60 | 0.7 | 0.83 |
| Species | s__sp. HMT-300.128 | NA |  | 0.62 | NA | 0.81 |  |
| Species | s__sp. HMT-308.28 | 0.26 | 0.82 | 0.40 | 0.84 | 0.91 | 0.54 |
| Species | s__sp. HMT-322.6 | 1.09 | 0.15 | 0.84 | 1.53 | 0.66 | 0.38 |
| Species | s__sp. HMT-346.43 | -0.06 | 0.97 | -0.87 | 0.24 | 0.78 | 0.9 |
| Species | s__sp. HMT-346.45 | 0.19 | 0.89 | -1.17 | 1.42 | 0.6 | 0.38 |
| Species | s__sp. HMT-417.45 | -0.03 | 0.98 | -0.34 | NA | 0.93 |  |
| Species | s__sp. HMT-448.13 | 0.04 | 0.96 | -0.39 | NA | 0.88 |  |
| Species | s__sp. HMT-448.15 | 0.30 | 0.79 | -0.54 | NA | 0.87 |  |
| Species | s__sp. HMT-448.17 | -0.39 | 0.82 | -1.23 | 0.54 | 0.7 | 0.83 |
| Species | s__sp. HMT-525.24 | -0.47 | 0.72 | -1.03 | 0.62 | 0.6 | 0.81 |
| Species | s__sp. HMT-807.63 | -1.17 | 0.15 | -2.97 | -0.23 | 0.12 | 0.9 |
| Species | s__sp. HMT-807.67 | -0.03 | 0.98 | -0.28 | 0.39 | 0.96 | 0.84 |
| Species | s__sp. HMT-897.22 | NA |  | -0.01 | NA | 1 |  |
| Species | s__sputigena.29 | -0.60 | 0.51 | NA | -0.16 |  | 0.92 |
| Species | s__sputigena.34 | NA |  | NA | 0.40 |  | 0.82 |
| Species | s__sputigena.45 | -0.24 | 0.84 | NA | -0.63 |  | 0.73 |
| Species | s__sputigena.5 | NA |  | -0.16 | NA | 0.98 |  |
| Species | s__sputigena.50 | -0.30 | 0.82 | -0.18 | -0.01 | 0.98 | 1 |
| Species | s__sputigena.65 | 0.29 | 0.82 | -0.35 | 1.51 | 0.93 | 0.44 |
| Species | s__sputigena.68 | -0.19 | 0.9 | -0.48 | 0.94 | 0.93 | 0.71 |
| Species | s__stomatis.38 | 0.64 | 0.44 | 0.42 | 1.16 | 0.9 | 0.42 |
| Species | s__tannerae.54 | 0.23 | 0.87 | 0.34 | 0.79 | 0.93 | 0.72 |
| Species | s__uli.16 | -0.68 | 0.56 | -1.59 | -0.17 | 0.46 | 0.93 |
| Species | s__veroralis.9 | 1.32 | **0.03** | 1.24 | 1.89 | 0.58 | 0.11 |
| Species | s__vincentii.17 | -1.04 | 0.38 | -1.03 | -1.22 | 0.74 | 0.52 |
| Species | s__vincentii.9 | -1.88 | **0.034** | -1.68 | -1.91 | 0.51 | 0.33 |
| Species | s__wadei.22 | -0.80 | 0.53 | -0.47 | -1.17 | 0.93 | 0.52 |
| Species | s__wadei.28 | 0.54 | 0.67 | 0.37 | 0.03 | 0.93 | 0.99 |
| Species | s__watanabei.3 | -2.08 | **0.03** | -1.51 | -1.97 | 0.58 | 0.37 |
| Species | s__wiggsiae.26 | 0.24 | 0.87 | -0.85 | 1.97 | 0.66 | 0.37 |

“NA, not available/not estimable; blank cells indicate that no FDR-adjusted p-value was calculated.”
