# Supplementary material for: The Causal Effect of Supra‐ and Sub‐Gingival Instrumentation on the Oral Microbiome of Elderly Periodontitis Patients Treated in Nursing Homes: Secondary Analysis of a Randomised Controlled Trial
Source: J Clin Periodontol. 2026 Aug 19;53(12):1823–31. doi: 10.1111/jcpe.70193 (PMC13751316; doi:10.1111/jcpe.70193)
Supplement: Supplementary file 2 [file JCPE-53-1823-s002.docx]

**Supplementary Methods.**

**The causal effect of supra- and subgingival instrumentation on the oral microbiome of elderly periodontitis patients treated in nursing homes: secondary analysis of a randomized controlled trial**

*Detailed Methods only available online.*

Anna Greta Barbe^1*^, Daniel Hagenfeld^2*^, Carolin Walther^1^, Dalia Beck^1^, Fedja Farowski^3,4^, Maria Vehreschild^3,4,5^, Sonja H. M. Derman^1^, Sven Kleine Bardenhorst^1,6^, Dirk Bleiel^1,6^

*shared first authorship

^1^Faculty of Medicine and University Hospital Cologne, Polyclinic for Operative Dentistry and Periodontology, University of Cologne, Cologne, Germany.

^2^Department of Periodontology and Operative Dentistry, Münster University Hospital, Münster, Germany

^3^Department I of Internal Medicine, University Hospital Cologne, Cologne, Germany.

^4^Goethe University Frankfurt, University Hospital Frankfurt, Department II of Internal Medicine, Infectious Diseases, Frankfurt am Main, Germany

^5^German Centre for Infection Research, partner site Bonn-Cologne, Germany

^6^Department of Clinical Epidemiology, Institute of Epidemiology and Social Medicine, University of Münster, Münster, Germany

^7^Joint dental practice Dres. Pia und Dirk Bleiel, Rheinbreitbach, Germany.

Study design and ethical approval

This microbiome investigation was conducted as a secondary analysis embedded within a previously registered randomized controlled clinical trial that evaluated the effectiveness of outreach-based non-surgical periodontal therapy (NSPT) among nursing home residents requiring domiciliary dental care. The parent clinical trial (Barbe et al., 2025) was approved by the Ethics Committee of the University of Cologne (reference number 22-1219) and registered in the German Clinical Trials Register (DRKS00029392). The present microbiome study adhered to the same ethical and methodological framework. All participants included in this analysis were enrolled in the parent trial and provided written informed consent for subgingival and supragingival microbial sampling. All study procedures were conducted in accordance with the Declaration of Helsinki and complied with relevant institutional and national ethical standards governing research involving human participants.

Study population

Participants were nursing home residents aged ≥65 years who met the inclusion criteria for outreach periodontal care in Germany. Key inclusion criteria were a diagnosis of periodontitis with probing pocket depths ≥4 mm, the absence of antibiotic therapy within the previous six months, and medical stability sufficient to permit dental examination and instrumentation. Exclusion criteria included conditions precluding safe periodontal instrumentation or anticipated short survival. Randomization and treatment allocation followed the procedures of the parent clinical trial.

Intervention

Periodontal treatment was delivered using an outreach-based NSPT protocol adapted for frail or care-dependent older adults residing in nursing homes. The intervention was performed on-site using a portable dental unit under practical conditions of domiciliary care. Patients were treated either seated in a wheelchair or supine in bed, as clinically feasible, with use of headlamps and mobile suction systems to optimize visibility and field control.

The NSPT procedure consisted of supra- and subgingival instrumentation of all accessible teeth using hand curettes and ultrasonic scalers, followed by polishing and pocket irrigation with 0.2% chlorhexidine (according to statutory treatment guidelines in Germany). The approach on-site in-room focused on the removal of soft and hard deposits without prior professional prophylaxis or staged pre-treatment, reflecting the limitations and logistical requirements of in-facility care. At baseline, full-mouth periodontal parameters were recorded, including bleeding on probing (BOP), plaque index (PI), and probing pocket depth (PPD). Supragingival and subgingival microbiome samples were collected from predefined sites immediately before treatment. The intervention group received the NSPT at baseline, with follow-up examinations after three months, during which clinical parameters and microbial samples were re-assessed using the same protocol. Participants assigned to the control group underwent identical baseline and three-month assessments without active periodontal treatment during the observation period. After completion of follow-up, all control participants were offered NSPT in accordance with ethical requirements. This standardized single-session NSPT-based outreach model aimed to evaluate the feasibility and short-term biological response to periodontal debridement under real-world domiciliary conditions in nursing home residents.

Clinical examination

At baseline and after three months, participants underwent a standardized periodontal examination conducted in the nursing home by the same calibrated investigator (DiBl). The following parameters were recorded at two sites per tooth using a periodontal probe (PCP 15, Hu‑Friedy, Chicago, IL, USA): BOP, PI, and PPD.

Plaque sampling

Subgingival and supragingival plaque samples were collected from all participants at baseline and at the three-month follow-up. Sampling sites were defined at baseline as the teeth exhibiting the deepest periodontal pockets in each quadrant and were kept constant for longitudinal sampling, irrespective of changes in PPDs over time. Supragingival biofilm samples were collected using a sterile curette. Subgingival plaque samples from the corresponding tooth were collected via sterile ISO 25 paper points (VDW GmbH, Munich, Germany). Paper points were inserted into the deepest periodontal pockets (PPD ≥4 mm) for 15 s and pooled (4 paper points/participant). Each sample was immediately transferred into sterile microtubes containing transport medium (DNA/RNA Shield, Zymo Research Corporation, USA) and stored at −80°C until DNA extraction.

DNA extraction and 16S rRNA gene sequencing

Genomic DNA was extracted from swabs stored in Zymo DNA/RNA Shield using the ZymoBIOMICS DNA Miniprep Kit (Zymo Research, Irvine, CA, USA). As per manufacturer’s protocol, the paper points or curette scrapings were transferred directly into the ZR BashingBead Lysis Tube and processed along with the storage medium. Concentration and quality of the extracted DNA was assessed spectrophotometrically (Nanodrop One, Thermo Fisher Scientific Inc., Waltham, MA, USA) and the amount of double-stranded DNA was quantified using the Qubit dsDNA kit (Thermo Fisher Scientific, Waltham, MA, USA). The V3-V4 region of the bacterial 16S rRNA gene was sequenced at an external service provider (Biomarker Technologies GmbH, Münster, Germany) using the 338F (5′-ACTCCTACGGGAGGCAGCA-3′) and 806R (5′-GGACTACHVGGGTWTCTAAT-3′) primers on an Illumina NovaSeq platform (paired-end 2 x 250bp). Sequencing data was processed using the DADA2 pipeline and QIIME version 2. Raw reads were trimmed and processed by the QIIME DADA2 plugin with the denoise-paired option and standard parameters. Taxonomic classification was performed by a Naïve Bayes classifier (sklearn), trained on the extended human oral microbiome database (eHOMD, 16S rRNA RefSeq Version 16.01). Raw reads are publicly available in the European Nucleotide Archive (ENA) under accession PRJEB105287.

Statistical analysis

Power calculation

To test how reliably our pipeline detected the group × visit interaction, we performed a parametric bootstrap based on the fitted negative-binomial GLMM, described below. We treated that model as the data-generating process, then simulated 150 synthetic datasets for each candidate interaction effect (log-fold changes 0.2, 0.4, 0.6, 0.8, 0.9, 1.0). Each dataset preserved the original design matrix, library-size offsets, dispersion, and subject-specific random intercepts. For every simulated dataset, we reran the full differential-abundance workflow; that is, taxon filtering, GLMM fitting, and Benjamini-Hochberg adjustment per rank and recorded whether the group × follow-up term remained significant (BH < 0.05). The empirical power curve (Suppl. Fig. 1) shows that our pipeline has <40 % power for log-effects <0.6, ~70 % power at log β = 0.8, and ≈85 % power for log-effects ≥0.9, well within the range of the major interaction shifts we observed.

**Supplementary Figure 1. Parametric bootstrap power curve for detecting the group × follow-up interaction in the differential-abundance pipeline.** Power was estimated by simulating 150 datasets per candidate interaction effect (log β), using the fitted (zero-inflated) negative-binomial mixed model as the data-generating process while preserving the original study design matrix, library-size offsets, dispersion, and subject-level random intercepts. For each simulated dataset, the full workflow (taxon filtering, GLMM fitting, and Benjamini-Hochberg correction within taxonomic rank) was rerun, and power was defined as the proportion of simulations in which the group × follow-up term remained significant (BH q < 0.05).

Clinical variables

Clinical, behavioral, and demographic characteristics were summarized by study arm (intervention vs. control). Between-group differences at baseline were assessed using the Mann-Whitney U test for continuous and ordinal variables and the Pearson χ² test for categorical variables. Analyses of clinical periodontal parameters (BOP, PI, PPD) were performed to confirm comparability with the parent clinical trial and assess short-term treatment effects. Descriptive statistics (mean ± standard deviation, range) were computed, with between-group differences tested using the Mann-Whitney U test and within-group changes evaluated using the Wilcoxon signed-rank test. Subgroup analyses by tooth type and corresponding effect sizes were additionally calculated. Those analyses were conducted using SPSS Statistics 28.0 (IBM Corp., Armonk, NY, USA) and for the microbiome analyses described below with R (R Foundation for Statistical Computing, Vienna, Austria; 2023), with statistical significance set at p < 0.05.

Longitudinal mixed-effects model specifications

All microbiological endpoints shared the same regression structure:

outcome ~ location + group × timepoint + library-size term + (1 | subject).

Here, the *group* contrasted Intervention vs Control, the *timepoint* contrasted follow-up with baseline, and the *location* adjusted for the sampling niche (supragingival/subgingival). Repeated measures were modeled with a subject-specific random intercept. Library size was included as a log(total reads) adjustment: for Gaussian endpoints, it was entered as a covariate; for count endpoints, it was entered as an offset(log(total reads)). This specification tested the group × timepoint interaction as the primary effect, as the **main endpoint of this study**.

Dysbiosis and alpha diversity

The Subgingival Microbial Dysbiosis Index (SMDI) was computed at the genus level as described by Chen et al.(Chen, Marsh, & Al-Hebshi, 2022). Standard alpha-diversity indices (Richness, Shannon, Simpson) were computed with mia::estimateDiversity on (Borman T, 2026) rarefied library sizes of 23,323 reads using phyloseq::rarefy_even_depth (McMurdie & Holmes, 2013). SMDI and alpha diversity were analyzed with linear mixed-effects models (nlme::lme, REML) (Pinheiro J, 2025) using the specification above.

Beta diversity

Bray-Curtis dissimilarities were computed on paired baseline and follow-up samples within each subject and location to capture within-person change. Because PERMANOVA cannot include random effects, we used an analogous fixed-effects design in vegan::adonis2 (Jari Oksanen [aut, 2025) that tested the group × timepoint interaction while adjusting for *location* and log(read depth). Subject-level dependence was handled via restricted, subject-blocked permutations (vegan::how(blocks = subject)), ensuring that the interaction term reflected differential trajectories between trial arms. For visualization, we computed a constrained ordination (vegan::capscale) using the same formula as the PERMANOVA, with subject-blocked permutations (blocks = subject).

Differential abundance modeling

We restricted analyses to taxa present in ≥20% of samples and with ≥100 reads to reduce zero inflation, improve model convergence, and maintain power under false discovery rate (FDR) control. A sensitivity run including rarer taxa (≥10% prevalence) nearly doubled the number of tests and produced more convergence warnings without altering the main conclusions, while lowering per-test power. Counts were modeled taxon-wise at each rank using glmmTMB::glmmTMB (Friedman, Hastie, & Tibshirani, 2010; Tay, Narasimhan, & Hastie, 2023) with a negative-binomial mean (family = nbinom2), a zero-inflation component (ziformula ~ 1), and the offset(log(total reads)); fixed and random effects according to the model specification. The group × timepoint coefficient yielded the log fold-change for Intervention vs Control at follow-up, adjusted for baseline and niche. Estimates were extracted with broom.mixed, Benjamini-Hochberg FDR correction was applied within each taxonomic rank, and significant lineages were visualized with MicroViz (David J.M. Barnett, 2021) taxonomic tree plots. For sensitivity analyses, we repeated the same specification within each niche (supragingival- and subgingival-only) by omitting the *location* term. Microbiota analyses were carried out using R version 4.5.1 (R Foundation for Statistical Computing, Vienna, Austria).

Supplementary Methods Literature

Barbe, A. G., Beck, D., Hellmich, M., von Kohout, M., Derman, S. H. M., & Bleiel, D. (2025). Shortened outreach periodontal therapy in nursing home residents with periodontitis: A randomized controlled trial. *J Periodontol*. doi:10.1002/jper.70041

Borman T, E. F., Shetty S, Lahti L. (2026). mia: Microbiome analysis. Retrieved from <https://microbiome.github.io/mia/>

Chen, T., Marsh, P. D., & Al-Hebshi, N. N. (2022). SMDI: An Index for Measuring Subgingival Microbial Dysbiosis. *J Dent Res, 101*(3), 331-338. doi:10.1177/00220345211035775

David J.M. Barnett, I. C. W. A., and and John Penders. (2021). microViz: an R package for microbiome data visualization and statistics. *Journal of Open Source Software*.

Friedman, J. H., Hastie, T., & Tibshirani, R. (2010). Regularization Paths for Generalized Linear Models via Coordinate Descent. *Journal of Statistical Software, 33*(1), 1 - 22. doi:10.18637/jss.v033.i01

Jari Oksanen [aut, c., Gavin L. Simpson [aut], F. Guillaume Blanchet [aut], Roeland Kindt [aut], Pierre Legendre [aut], Peter R. Minchin [aut], R.B. O'Hara [aut], Peter Solymos [aut], M. Henry H. Stevens [aut], Eduard Szoecs [aut], Helene Wagner [aut], Matt Barbour [aut], Michael Bedward [aut], Ben Bolker [aut], Daniel Borcard [aut], Tuomas Borman [aut], Gustavo Carvalho [aut], Michael Chirico [aut], Miquel De Caceres [aut], Sebastien Durand [aut], Heloisa Beatriz Antoniazi Evangelista [aut], Rich FitzJohn [aut], Michael Friendly [aut], Brendan Furneaux [aut], Geoffrey Hannigan [aut], Mark O. Hill [aut], Leo Lahti [aut], Cameron Martino [aut], Dan McGlinn [aut], Marie-Helene Ouellette [aut], Eduardo Ribeiro Cunha [aut], Tyler Smith [aut], Adrian Stier [aut], Cajo J.F. Ter Braak [aut], James Weedon [aut]. (2025). Community Ecology Package. Retrieved from <https://vegandevs.github.io/vegan/>, <https://github.com/vegandevs/vegan>

McMurdie, P. J., & Holmes, S. (2013). phyloseq: An R Package for Reproducible Interactive Analysis and Graphics of Microbiome Census Data. *PLOS ONE, 8*(4), e61217. doi:10.1371/journal.pone.0061217

Pinheiro J, B. D., R Core Team. (2025). nlme: Linear and Nonlinear Mixed Effects Models. Retrieved from <https://CRAN.R-project.org/package=nlme>

Tay, J. K., Narasimhan, B., & Hastie, T. (2023). Elastic Net Regularization Paths for All Generalized Linear Models. *Journal of Statistical Software, 106*(1), 1 - 31. doi:10.18637/jss.v106.i01
