# Supplementary material for: The Causal Effect of Supra‐ and Sub‐Gingival Instrumentation on the Oral Microbiome of Elderly Periodontitis Patients Treated in Nursing Homes: Secondary Analysis of a Randomised Controlled Trial
Source: J Clin Periodontol. 2026 Aug 19;53(12):1823–31. doi: 10.1111/jcpe.70193 (PMC13751316; doi:10.1111/jcpe.70193)
Supplement: Supplementary file 3 [file JCPE-53-1823-s003.docx]

**Supplementary Table 2.** Sensitivity analysis: Baseline-adjusted ANCOVA model for follow-up Subgingival Microbial Dysbiosis Index (SMDI).

ANCOVA-Model: followup_smdi ~ treatment_group + baseline_smdi
N: Waiting list = 17, Intervention = 18

| **Term** | **Estimate** | **SE** | **t** | **p** | **95% CI** |
| --- | --- | --- | --- | --- | --- |
| Intercept | 1.047 | 0.511 | 2.051 | 0.0485 | 0.007 to 2.087 |
| Intervention vs Waiting list | -1.940 | 0.719 | -2.698 | 0.0110 | -3.405 to -0.476 |
| Baseline SMDI | 0.773 | 0.137 | 5.645 | 3.04e-06 | 0.494 to 1.052 |

Residual SE = 2.053 on 32 df; R² = 0.511; adjusted R² = 0.481; model p = 1.06e-05.
